# New BDNF and NT-3 Cyclic Mimetics Concur with Copper to Activate Trophic Signaling Pathways as Potential Molecular Entities to Protect Old Brains from Neurodegeneration

**DOI:** 10.3390/biom14091104

**Published:** 2024-09-02

**Authors:** Antonio Magrì, Barbara Tomasello, Irina Naletova, Giovanni Tabbì, Warren R. L. Cairns, Valentina Greco, Sebastiano Sciuto, Diego La Mendola, Enrico Rizzarelli

**Affiliations:** 1 Institute of Crystallography, National Council of Research (CNR), P. Gaifami 18, 95126 Catania, Italy; antonio.magri@cnr.it (A.M.); irina.naletova@ic.cnr.it (I.N.); giovanni.tabbi@cnr.it (G.T.); 2 Department of Drug and Health Sciences, University of Catania, Viale Andrea Doria 6, 95125 Catania, Italy; btomase@unict.it; 3 CNR-Institute of Polar Sciences (CNR-ISP), 155 Via Torino, 30172 Venice, Italy; warrenraymondlee.cairns@cnr.it; 4 Department of Chemical Sciences, University of Catania, Viale Andrea Doria 6, 95125 Catania, Italy; vgreco@unict.it (V.G.); ssciuto@unict.it (S.S.); 5 Department of Pharmacy, University of Pisa, via Bonanno Pisano 6, 56126 Pisa, Italy; diego.lamendola@unipi.it

**Keywords:** neurotrophines, metallostasis, copper signaling, copper complexes, peptide mimetics

## Abstract

A low level of Neurotrophins (NTs), their Tyrosine Kinase Receptors (Trks), Vascular Endothelial Growth Factors (VEGFs) and their receptors, mainly VEGFR1 and VEGFR2, characterizes AD brains. The use of NTs and VEGFs as drugs presents different issues due to their low permeability of the blood−brain barrier, the poor pharmacokinetic profile, and the relevant side effects. To overcome these issues, different functional and structural NT mimics have been employed. Being aware that the N-terminus domain as the key domain of NTs for the binding selectivity and activation of Trks and the need to avoid or delay proteolysis, we herein report on the mimicking ability of two cyclic peptide encompassing the N-terminus of Brain Derived Growth Factor (BDNF), (c-[HSDPARRGELSV-]), cBDNF(1-12) and of Neurotrophin3 (NT3), (c-[YAEHKSHRGEYSV-]), cNT3(1-13). The two cyclic peptide features were characterized by a combined thermodynamic and spectroscopic approach (potentiometry, NMR, UV-vis and CD) that was extended to their copper(II) ion complexes. SH-SY5Y cell assays show that the Cu^2+^ present at the sub-micromolar level in the complete culture media affects the treatments with the two peptides. cBDNF(1-12) and cNT3(1-13) act as ionophores, induce neuronal differentiation and promote Trks and CREB phosphorylation in a copper dependent manner. Consistently, both peptide and Cu^2+^ stimulate BDNF and VEGF expression as well as VEGF release; cBDNF(1-12) and cNT3(1-13) induce the expression of Trks and VEGFRs.

## 1. Introduction

Alzheimer’s disease (AD) is one of the main neurodegenerative diseases of the central nervous system in elderly people [1]. The major hallmarks of AD are soluble toxic amyloid-β (Aβ) peptide oligomers [2], which are proteolytic fragments of the amyloid precursor protein (APP) cleavage by β-secretases and γ-secretases [3], neurofibrillary tangles (NFTs) primarily composed of hyperphosphorylated tau proteins [4], selective basal forebrain cholinergic neuron (BFCN) degeneration and brain atrophy [5]. Amongst the various hypotheses for AD pathogenesis, the amyloid cascade hypothesis has prevailed in the last few decades [6,7].

Since 1981 another explanation for the pathogenesis of AD has been proposed, named the neurotrophic factor hypothesis [8,9] represented by the nerve growth factor (NGF) hypothesis [10,11], according to which the altered neurotrophic status, usually supported by NGF and other neurotrophic factors (NTFs), affects AD progression [12,13]. As both amyloidosis and NTFs are involved in AD pathogenesis, adjusting their neurotoxic and neurotrophic effects should be helpful to avoid AD development. Consistently, different studies have reported the neurotoxic effects of Aβ oligomers and the neurotrophic roles of NTFs on AD pathogenesis [4,14,15,16,17], showing that the signaling pathways of NTFs can modulate amyloidogenesis [18,19], and how proteins involved in the amyloidogenic processes can influence the NTF signals [20]. These proteins consist of four following families: neurotrophins, neuropoietic cytokines, glial cell line-derived NTF (GDNF) ligands (GFLs), and cerebral dopamine neurotrophic factor-mesencephalic astrocyte-derived neurotrophic factor (CDNF/MANF) [21].

Neurotrophins (NTs) include nerve growth factor, brain-derived neurotrophic factor (BDNF), neurotrophin-3 (NT3), and neurotrophin-4/5 (NT-4/5); they drive different cellular processes in the central (CNS) and peripheral nervous systems (PNS) [22]. These diverse biological roles are determined by their interactions with one of two receptors: tyrosine receptor kinase (Trk), a member of the tropomyosin-related tyrosine kinase receptors, or p75 neurotrophin receptor (p75NTR), a member of the tumor necrosis factor (TNF) receptor superfamily [23]. Pro-neurotrophins are first synthesized and bind to their receptor p75NTR. Cleavage of pro-neurotrophins generates mature neurotrophins, which bind selectively one of three types of tyrosine kinase (Trk) receptors [23]. When neurotrophins interact with specific Trk receptors (NGF binds to TrkA, BDNF and NT4/5 bind to TrkB, and NT3 binds to TrkC and with lesser affinity to the other Trks), accelerated downstream cell signaling occurs [24]. Moreover, their interaction with p75NTR leads to the modulation of brain plasticity and apoptosis [25]. In CNS, BDNF results as the most prominent neurotrophin due to the wide TrkB expression in the brain, mainly in the cortex, hippocampus, and basal forebrain [26].

Postmortem brain district analyses of patients with AD show regional specific differences on the levels of NTs, such as NGF, BDNF and NTF-3 [27]. Their quantitative determination in AD mouse models display similar imbalances suggesting that these neurotrophins may be recognized therapeutic targets in AD [28,29]. Consistently, different studies demonstrate the major involvement of BDNF and NGF among NTs in AD pathology, showing significant variations in neurotrophins and their Trk receptors expression and signaling with altered neuroprotection, neuroplasticity, and cognitive function [16,30]. NGF deprivation induces Aβ accumulation/aggregation, while NGF administration improves Aβ pathologic changes in animal models [31] and upregulates the expression of α-secretase promoting APP cleavage towards the non-amyloidogenic pathway [32,33] and downregulates [34] the expression of β-site amyloid precursor protein cleaving enzyme, BACE1, responsible for the production of Aβ [35].

AD highlights the different role of TrkA and p75NTR. The aging pathway decreases TrkA expression, results in a TrkA-to p75NTR receptor switch for NGF signaling and leads to Aβ peptide generation, potentially explaining why aging is a risk factor for AD [36,37,38]. NGF/TrkA signaling activates microglia to promote microglia phagocytosis of soluble Aβ oligomers, which are rapidly transferred to lysosomes, thus enhancing the degradation of toxic Aβ oligomers [39].

BDNF is widely distributed throughout the CNS and is a critical component required for the function and survival of neurons [40,41]. AD has been the subject of extensive research on BDNF dysregulation and changed expression [42,43]. Two forms of BDNF are present in the nervous system: mature mBDNF and its precursor, proBDNF, both display a decrease trend at the beginning of AD and significantly reduce as the disease advances [44]. BDNF/TrkB signaling shows a critical role in mitigating AD symptoms [45], in slowing Aβ-induced neurodegeneration, sustaining neuron viability, and alleviating synaptic alterations, which can avoid cognitive damage [46,47]. BDNF binding to TrkB induces differentiation and maturation of adult-born neurons through GABAergic transmission, decreasing the damage due to the drop of neurogenesis and maturation reduction that characterize AD patients [48,49]. BDNF/TrkB performs many different protective effects by promoting the activation of several intracellular signal pathways, involving phospholipase C-γ (PLC-γ)/protein kinase C (PKC), mitogen-activated protein kinase (MAPK)/extracellular regulated kinase (ERK), namely MEK, and phosphoinositide 3-kinase (PI3K)/AKT signal pathways [50,51,52] and cAMP Response Element-Binding Protein (CREB). All these elements are relevant and critical players in the modulation of neurogenesis whose deficit characterizes AD [53].

While the huge number of investigations on the role of neurotrophins in the healthy and sick CNS are focused on BDNF and TrkB, it is well known that most neurons co-express the TrkB and TrkC receptors [54] showing that Neurotrophin-3 (NT3) may also play a role in improving cognitive functions in AD. NT3 binding to Trk-C modulates neurogenesis and favors hippocampal plasticity, suggesting a key role in memory function [55]. NT3 can also activate TrkB and downstream of Trk activation, the gene expression variations induced by TrkC activation result similar to those provoked by TrkB activation by BDNF, including some of those involved in synaptic plasticity [56]. Furthermore, NT3 preserves cortical neurons against Aβ-driven neural cell death by restricting caspase-8, caspase-9, and caspase-3 cleavage [57].

Vascular dysfunction plays a main role in the pathogenesis of AD [58], that is characterized by cerebral blood flow decrease [59] associated with poor cognitive performance [60]. Among the newly identified angiogenic modulators, several belong to the family encompassing those that are accepted as nerve growth factors [61,62]. In this context, different reports highlight the ability of NGF to induce angiogenesis in in vitro and in vivo [63], by a TrkA/MAPK/ERK -dependent pathway activation [64], while BDNF/TrkB activation provokes Vascular Endothelial Growth Factor, VEGF, expression via hypoxia-inducible factor-1alpha, HIF-α and tunes its secretion [65,66]. Moreover, NT3 behaves as an angiogenic mediator inducing neovascularization in a mouse model of ischemia, highlighting the dynamic connection between the neuronal and vascular system. VEGFs belong to a family of structurally related homodimeric proteins, which encompass VEGF-A, -B, -C, -D, and placental growth factor in mammals [67,68]. VEGFs transfer their signals by stimulating their specific tyrosine kinase receptors (VEGFRs), i.e., VEGFR-1 or fms-like tyrosine kinase-1 (Flt-1), VEGFR-2 or kinase insert domain-containing receptor (Kdr), and VEGFR-3 or Flt-4 [69]. Alteration of VEGFs/VEGFRs pathways and consequent abnormal angiogenesis affect different diseases [70,71]. while VEGF-A treatment of AD hippocampal neurons cultures, by regularizing VEGFR-2 signaling, partly recovers the Aβ-induced variations of dendritic spine morphology and density impairments [72]. A reduction of VEGFR-2 mRNA amount is found in Aβ-exposed HUVECs, decreased cerebral VEGFR-2 protein levels characterize some transgenic mouse AD models [73]. Aβ peptide oligomers can also directly lead to cerebrovascular alterations, as they accumulate around and within the cerebral vessel walls [74,75], inhibiting angiogenesis [76]. However, VEGF avoids Aβ-induced endothelial apoptosis in vitro and its neuronal expression in transgenic mice recovers memory alterations [77]. Independent of changes in VEGF-A levels, the microvascular perturbation is suggested to occur in AD by direct Aβ-VEGFR-2 interaction, that counteracts VEGF-A-induced receptor activation [78], or by VEGF-A segregation in Aβ fibrillar aggregates [79], a process that starts at the beginning of the pathology and enhances when AD progress [80].

In summary, NTs play crucial roles in the prevention and treatment of AD [81] and therapeutic uses of neurotrophins by emergent delivery systems are reported in clinical trials [82,83]. Although these clinical trials show that NGF specifically affects AD patients [84], there are significant draw backs such as pain, weight loss and issues to control the dose [85]. Moreover, because all NTs are high molecular-weight proteins which cannot easily cross the blood-brain barrier, they promote immune responses and are not stable under physiological conditions, showing short half-life in plasma and there is a lack of them in sufficient amounts for clinical use [86,87]. Furthermore, VEGF or VEGFR regulation is not considered a viable approach to treat AD due to their multiple and complex effects in brain homeostasis [88].

The recent findings in neurotrophin structure and their binding receptors, favors now the design and development of small molecules that mimic NT activities and induce the synthesis of endogenous neurotrophic factors [89]. Among functional and structural NT mimics, peptide design and development appear to play a main role [90,91,92]. Consistently with the experimental and computational findings that indicate the N-terminus domain as the key domain of NTs for the binding selectivity and activation of Trks [93,94], new synthesized linear monomeric peptides encompassing the 1-14 residues of NGF (NGF 1-14) [95,96], the 1-12 residues of BDNF (BDNF 1-12) [97], the 1-13 residues of NT-3 (NT3(1-13) [98] and two dimeric peptides (d-NGF1-15) [99] and (d-BDNF1-13) [100] show some typical neurotrophin features. The sequence specific effects of these NT mimetics include the activation of the MAPK/ERK1/2 and PI3K/AKT signalling pathways, with the consequent CREB phosphorylation, BDNF expression and release as well as neurite outgrowth [99]. Furthermore, these NT mimicking peptides bind Cu^2+^ and Zn^2+^ and are shown to be efficient ionophores [96,99], increasing metal intracellular bioavailability that affects the neurotrophic-induced signaling cascade [101]. More recently, a study reports on the 14-membered cyclic peptide, reproducing the NGF N-terminus (SSSHPIFHRGEFSV (c-NGF(1-14), ability to: (i) significantly delay its degradation by proteolytic enzymes, (ii) induce cell differentiation, (iii) mimic the protein in TrkA and VEGF activation (iv) promote the BDNF and VEGF expression and release [102].

Here we report on the thermodynamic and spectroscopic characterization (NMR, UV-vis and CD) of the twelve membered cyclic peptide, reproducing the N-terminus sequence of BDNF (c-[HSDPARRGELSV-]), cBDNF(1-12) and the thirteen membered analogue, representing the N-terminal sequence of NT3 (c-[YAEHKSHRGEYSV-]), cNT3(1-13). The histidine residues present in the cyclic peptides favor the copper(II) ion binding with significant affinity; the resulting metal complexes are characterized by potentiometric and spectroscopic measurements. Both the cell culture media and the supplemented serum copper content was determined by ICP-OES. Experiments on SH-SY5Y cells show that the Cu^2+^ present at the sub-micromolar level in the complete culture media affects the treatments with the two peptides, whose effects are further enhanced by metal ion addition (10 μM). cBDNF(1-12) and cNT3(1-13) act as ionophores, inducing the neuronal differentiation of neuroblastoma cells and promoting Trk signaling cascade by CREB phosphorylation in a copper dependent manner. Consistently, both peptide and Cu^2+^ stimulate BDNF and VEGF expression as well as VEGF release; cBDNF(1-12) and NT3(1-13) induce the expression of Trks and VEGFRs.

## 2. Materials and Methods

### 2.1. Design and Synthesis

The design of cyclic peptides was inspired by our previous work on the parent linear peptides [97,98], while the cBDNF(1-12) and cNT3(1-13) were synthesized by CASLO (Kongens Lyngby, Denmark). All other chemicals, of the highest available grade, were purchased from Sigma-Aldrich (Munich, Germany) and used without further purification.

### 2.2. NMR Measurements

^1^H NMR spectra were recorded by a 500 MHz Varian Unity Inova spectrometer. Experiments were carried out in D_2_O at 27 °C at an 8 mg/mL concentration. Chemical shifts are reported as δ (ppm) and are referenced to the residual HOD resonance. Unequivocal assignments of 1H resonances were supported by gCOSY experiments. The VnmrJ v4.0 software was used to process the data.

### 2.3. Potentiometric Titrations

Potentiometric titrations were performed on a Titrando 905 automatic titrator (Herisau, Switzerland) using a combined glass-Ag/AgCl electrode (Metrohm, Switzerland). The titration cell (2.5 mL) was thermostated at 298.0 ± 0.2 K, and all solutions were kept under an argon atmosphere. KOH solutions (0.1 M) were added through a Dosino 800 burette (Metrohm, Switzerland) equipped with 2 cm^3^ syringe. The ionic strength of all solutions was adjusted to 0.10 M (KNO_3_). For the determination of protonation and complexation constants, peptide concentrations ranged from 5.0 to 7.5 × 10^−4^ M. A minimum of three independent titrations were performed for each system in the 2.4–11.0 pH range. Metal-to-ligand ratio of 1:1 was employed. To avoid systematic errors and verify reproducibility, the electromotive force (EMF) values of each experiment were taken at different time intervals. Other experimental details were as previously reported [103]. To obtain complexation constants, the potentiometric data were refined by using Hyperquad [104]. The species distribution as a function of the pH was obtained by using Hyss [105].

### 2.4. Ultraviolet-Visible (UV-Vis) Measurements

UV-vis spectra were recorded at 298.0 ± 0.2 K employing an Agilent 8453 (Agilent Technologies, Santa Clara, CA, USA) or a Jasco V-670 (Jasco Europe s.r.l., Cremella (LC), Italy) spectrophotometer. All solutions were freshly prepared by using twice-distilled water. The concentrations of the peptides and copper(II) ion used to record absorption spectra were the same as those employed for the potentiometric titrations. The results are reported as ε (molar adsorption coefficient) in M^−1^ cm^−1^.

Combined spectroscopic and potentiometric metal-complex titrations were performed in a 3 mL quartz cuvette with a 1 cm path length to obtain the spectrum in the visible region at each pH value simultaneously. These experiments were replicated at least three times for each copper-peptide system. Spectroscopic data were processed by means of Hyperquad program [104].

### 2.5. Circular Dichroism (CD) Measurements

CD spectra were obtained at 298.0 ± 0.2 K under a constant flow of nitrogen on a Jasco model 1500 spectropolarimeter (Jasco Europe s.r.l., Italy). CD spectra of the peptides were recorded in the 190–300 nm wavelength range (scan rate: 50 nm min^−1^, resolution: 0.1 nm, path length 1 cm, averaged scans 10–20), varying the pH value from 4 to 11. The peptide concentration was 5 × 10^−6^ M. The CD spectra of Cu^2+^ complexes were obtained in the 190–300 nm (scan rate: 50 nm min^−1^, resolution: 0.1 nm, path length 1 cm, averaged scans 10–20) and 250–750 nm wavelength ranges (scan rate: 100 nm min^−1^, resolution: 0.1 nm, path length 1 cm, averaged scans 3) by varying the pH from 4 to 11. The peptide concentrations were 5 × 10^−6^ M (in the Far-UV experiments) and 1 × 10^−3^ M (in the UV-vis measurements). A 1:1 molar ratio metal to ligand was used in both cases. All solutions were freshly prepared using double distilled water. The results are reported as Δε (molar dichroic coefficient) in M^−1^ cm^−1^.

### 2.6. Inductively Coupled Plasma—Optical Emission Spectroscopy

Cu was quantified using a Thermo Scientific iCAP 7400 Duo ICP-OES operating in Dual View mode with the polychromator under N_2_ purge to reduce molecular interferences from air. We used two analytical wavelengths (i.e., Cu 324.754 and Cu 327.396) to confirm the concentrations found were free from interferences. The calibration standards at 0, 1, 10, 50 and 100 µg/L were made by mass in polypropylene metal free centrifuge tubes to ensure low blanks and reproducibility. The calibrations had an excellent linearity and the instrumental detection limits (IDL) were sufficient to allow 5-fold sample dilution to reduce matrix effects. The diluent chosen to keep the proteins and metals stable in solution was a mix of Triton X-100 (0.1% *v*/*v*), ammonia (1% *v*/*v*) and EDTA (0.1% *m*/*v*) in ultra pure 18M ohm water (Elga Lab Water, High Wycombe, UK).

### 2.7. Cell Culture Reagents and Antibodies

For the cellular experiments, Dulbecco’s modified eagle medium DMEM/F12, Dulbecco’s modified eagle medium high glucose, penicillin-streptomycin solution, L-glutamine, fetal bovine serum (FBS), Dulbecco’s phosphate-buffered saline (PBS) and paraformaldehyde were purchased from Sigma-Aldrich (St. Louis, MO, USA).

The primary antibodies used for protein detection are as follows in Table 1:

The secondary goat anti-rabbit (Cat# 926-68071 and 925-32211) and goat anti-mouse (Cat# 926-68070 and 925-32210) labeled with IRDye 680 (1:20,000) and IRDye 800 (1:20,000), respectively, were from LI-COR (Lincoln, NE, USA). The secondary antibody for immunofluorescence assay was anti-rabbit IgG conjugated AlexaFluor 488 (Cat# A11008, 1:500 dilution, ThermoScientific, Waltham, MA, USA). Wheat Germ Agglutinin, Alexa Fluor 594 conjugate (Cat# 11262, 5 μg/mL, ThermoScientific, Waltham, MA, USA) was used to stain cellular membrane. Hoechst33342, and Halt Protease and Phosphatase Inhibitor Single-Use Cocktail was obtained from ThermoFisher (Waltham, MA, USA).

### 2.8. Cell Culture and Treatment Conditions

The SH-SY5Y cells were grown in DMEM/F12 supplemented with 10% fetal bovine serum (FBS), 2 mM glutamine and antibiotics in a humidified atmosphere of air/CO_2_ (95:5%) at 37 °C (Heraeus Hera Cell 150C incubator). For the treatments, cells were plated at a low density in the medium for differentiation (DM medium: DMEM high glucose with 0.5% FBS) on TPP^®^ tissue culture plates (Sigma-Aldrich, St. Louis, MO, USA), and after treated with cBDNF(1-12) or cNT3(1-13) (60 µM) with or without 10 µM Cu^2+^; for the cells treatment in the presence of 2,9-Dimethyl-4,7-diphenyl-1,10-phenanthroline disulphonic acid (BCS), DM medium was pre-treated with 50 μM solution of BCS for 24 h before adding to the cells cBDNF(1-12) or cNT3(1-13). The incubation time was chosen according to each experiment design, as described shortly hereafter.

### 2.9. Neuronal Differentiation of SH-SY5Y by Neurite Outgrowth Analysis

To determine the neurite growth, cells were seeded at a density of 1.5 × 10^4^ cells per well in 150 μL DM medium on 96 TPP^®^ tissue culture plates (Sigma-Aldrich, St. Louis, MO, USA) for 24 h. Cells were incubated with cBDNF(1-12) or cNT3(1-13) (100 µM) in presence or absence of 10 µM Cu^2+^ or 50 µM BCS in DM medium for 72 h; Hoechst 33342 (Thermo Fisher, Waltham, MA, USA) was then added to stain the nuclei; Wheat Germ Agglutinin, Alexa Fluor 594 conjugate (5 µg/mL, Thermo Fisher, Waltham, MA, USA) was used to stain cellular membranes. Four fields per well were then randomly examined under a Leica DMI 6000B epifluorescence inverted microscope with Adaptive Focus Control and quantitative analysis of the total neurite length was performed using the ImageJ software (v1.46d version, NIH). A Hoechst 33342 solution was used to normalize data to the actual cell number for each microphotograph.

### 2.10. Determination of Ctr1 Translocation by Immunofluorescence Assay

SH-SY5Y cells were seeded on 48-well plate, incubated for 24 h, maintained in DM medium overnight and finally treated by adding cNT3(1-13) or cBDNF(1-12) solutions (60 µM) containing either the peptide or the peptide with Cu^2+^ (10 µM) or BCS (50 µM) in DM medium (72 h). Cells were then fixed in 4% paraformaldehyde. Wheat Germ Agglutinin, Alexa Fluor 594 conjugate (5 µg/mL, Thermo Fisher, Waltham, MA, USA) and Hoechst33342 fluorescent dye (1 μg/μL) were used to stain cellular membranes and nuclear DNA, respectively. Unspecific binding was blocked by incubation in PBS with 0.2% gelatine for 30 min. Ctr1 plasma membrane level was detected by incubating non-permeabilized cells with rabbit anti-Ctr1 antibody overnight. After washing with PBS, cells were exposed at RT for 1 h to the secondary goat anti-rabbit IgG conjugated with AlexaFluor 488. Hoechst33342 fluorescent dye (1 μg/μL) was used to stain nuclear DNA. Images were analyzed with a Leica DMI 6000B epifluorescence inverted microscope with Adaptive Focus Control. Image analysis after anti-Ctr1 immunostaining was carried out using ImageJ Software 1.53 g (https://imagej.net/ij/ (accessed on 29 August 2024)); Java 1.8.0_112 (64bit).

### 2.11. Protein Lysate Preparation and Immunoblotting

Cells were exposure to cNT3(1-13) or cBDNF(1-12) (60 µM) solutions containing 10 µM CuSO_4_ or 50 µM BCS in DM medium for 10 min and 30 min to analyze Trk and CREB phosphorylation, respectively; 24 h and 72 h treatment was used for the analysis of VEGF and BDNF or Trk and VEGF receptors expression, respectively.

Sample preparation and western blot analysis were carried out according to the method described in a previous paper [102].

After the treatment, SH-SY5Y cells were collected by centrifugation at 1000× *g* at 4 °C for 5 min; cell pellets thus obtained were lysed in RIPA buffer (50 mM TRIS-HCl, pH 8.0, 150 mM NaCl, 0.5 mM EDTA, 1% Triton X-100, 0.5 mM EGTA, 1% NP40, 0.1% SDS) containing 0.5 mM EDTA, 1% Triton X-100, 0.5 mM EGTA and a Halt Protease and Phosphatase Inhibitor Single-Use Cocktail) for 30 min and then centrifuged at 14,000× *g* for 10 min. Total protein amount was determined by Bradford’s method (Protein Assay Dye Reagent Concentrate, BioRad, Hercules, CA, USA).

For western blot analysis, equal amounts of proteins were separated by 4–12% Tris-Glycine gels (Bio-Rad, Hercules, CA, USA) and transferred onto nitrocellulose membranes. Proteins were detected with specific primary antibodies reported in Table 1 by incubation overnight at 4 °C. The appropriate infrared-dye labeled secondary antibodies were used to detect primary antibodies.

The Odyssey Infrared Imaging System (LI-COR Biosciences, Lincoln, NE, USA) was used to scan the blot; quantitative densitometric analysis was performed by using ImageJ ((https://imagej.net/ij/ (accessed on 29 August 2024)); Java 1.8.0_112(64bit)). The results were expressed as arbitrary densitometric units (A.D.U.) and the values were normalized to either GAPDH or Actin expression levels as indicated. The level of phosphorylation was calculated as the ratio between the phosphorylated and unphosphorylated form of the protein.

### 2.12. Sandwich ELISA Assay

Medium samples were collected after a 24 h treatment exposure to cNT3(1-13) or cBDNF(1-12) (60 µM) solutions, respectively, containing 10 µM Cu^2+^ or 50 µM BCS in DM medium; samples were then centrifuged (14,000× *g*, 10 min) and supernatants were transferred into clean microtubes and stored at −80 °C until analyzed.

The concentration of VEGF released was determined from the cell culture media samples using the ELISA sandwich assay according to the method described previously. Polyvinyl chloride (PVC) microtiter plates were coated overnight at 4 °C with 5 µg/mL of capture antibody (anti-VEGF, code: PAB12284) in carbonate/bicarbonate buffer (pH 9.6). Then plates were washed twice with PBS, blocked by a blocking buffer (5% BSA/PBS) at room temperature for 2 h, washed with PBS and incubated with cell culture media samples at 37 °C for 90 min. Plates were then washed with PBS, incubated for 2 h with 1 µg/mL of detection antibody (anti-VEGF, code: H00007422-M05), washed again, incubated for 2 h with HP-conjugated secondary antibody and finally washed with PBS. After incubating for 15 min the amount of VEGF released was determined by using 3,3′,5,5′-tetramethylbenzidine (TMB) solution. The reaction was stopped by an appropriate solution (2 M H_2_SO_4_) and the optical density was measured at 450 nm by a plate reader (Varioskan^®^ Flash Spectral Scanning Multimode Reader, Waltham, MA, USA).

### 2.13. Statistical Analysis

All data are expressed as mean ± standard deviation of three experiments performed at least in triplicate. Analyses were performed using GraphPad Prism (GraphPadSoftware, San Diego, CA, USA) statistical software (version 5). The One-way ANOVA test, followed by Tuckey’s test was applied. A value of *p* < 0.05 was considered statistically significant.

## 3. Results

### 3.1. cBDNF(1-12) and cNT3(1−13) Are Cyclic Peptides Which Show Different Secondary Structures

^1^H NMR and Far-UV CD were employed to characterize cBDNF(1-12) and cNT3(1-13).

In the NMR spectrum of cBDNF(1-12), all the expected signals for the desired amino acid sequence are present (Figures S1 and S2).

Two signals are detectable in the aromatic region of the spectrum of cBDNF(1-12) at 8.68 and 7.36 ppm, which respectively correspond to those of the C-2 and C-5 protons of the imidazole ring of the histidine residue (Figure S1). The α-proton signals of all amino acid residues are detectable in the range of 5.04 to 3.50 ppm, and some of them partially overlap those of β-methylene of the serine residues and δ-methylene of the arginine ones. The β-methylene signal of the histidine residue as well as that of the δ-methylene of the proline residue fall in the region of the spectrum between 3.25 ÷ 3.00 ppm. The diastereotopic β-methylene protons of the aspartic acid residue appear as two signals at 2.83 and 2.75 ppm. The proton signals from arginine β- and *γ*-methylenes, leucine β- methylene, as well as those from β- and *γ*-methylenes of glutamic acid and proline all appear in the region between 1.15 and 2.25 ppm and are partially superimposed on leucine *γ*-methine and valine β-methine signals. Finally, the methyl signal of alanine appears at 1.40 ppm, while methyl groups of valine and leucine are detected as two doublets at 0.98 and 0.94 ppm, respectively. The relative ratios between the integration areas of the signals in the spectrum matches the expected amino acid composition for cBDNF(1-12).

Also, the ^1^H NMR spectrum of cNT3(1-13) displays all the signals consistent with the desired amino acid composition of the peptide (Figure S2). In the aromatic region of the spectrum (9.00 ÷ 6.00 ppm), the presence of two distinct groups of signals can be observed. The C-2 and C-5 proton signals of the imidazole ring of the two histidine residues appear at 8.63 and 7.31 ppm, respectively, while the phenyl ring proton signals of the tyrosine residues are observed at 7.12 (C-2, C-6) and 6.82 (C-3, C-5) ppm. The signals from the *α*-protons of all the amino acid residues are detected in the 4.78 ÷ 3.70 ppm range and overlap with the β-methylene signals of the serine residues and the *δ*-methylene signal of the arginine residue. The β -methylene signal of the histidine residues is detected together with tyrosine β-methylene and lysine ε-methylene residues at 3.38 ÷ 2.85 ppm. The proton signals of arginine β- and *γ*-methylenes, as well as glutamic acid β- and *γ*-methylenes and lysine β-, γ- and δ-methylenes, all appear in the region between 2.45 ÷ 1.40 ppm and are partially superimposed on valine β-methine signals. Finally, the methyl signals of alanine and valine are detected as two distinct doublets at 1.367 and 0.80 ppm respectively. The relative integration areas of all proton signals in the spectrum are also consistent with the amino acid composition of the cyclic peptide.

**Figure 1:**
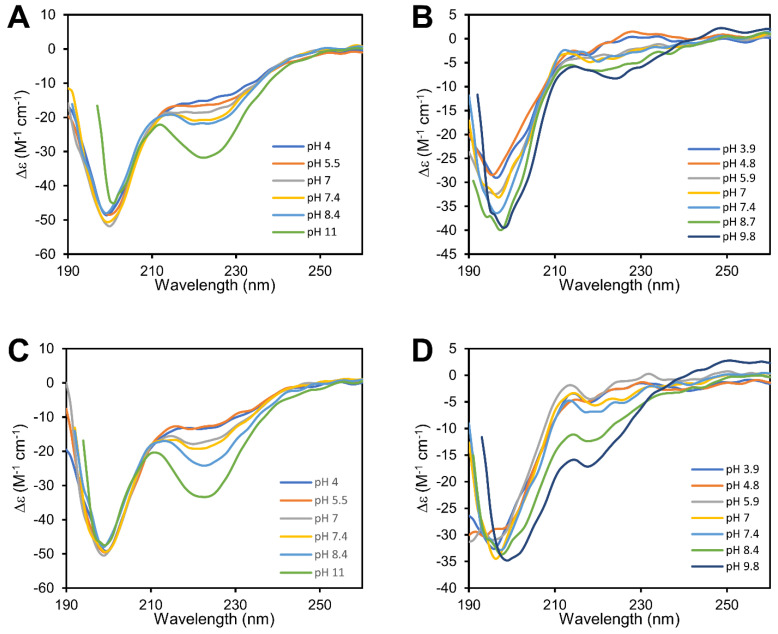
Far UV-CD spectra of (**A**) cBDNF(1-12), (**B**) cNT3(1-13), (**C**) Cu-cBDNF(1-12) and (**D**) Cu-cNT3(1-13) at different pH values ([L] = 5 × 10^−6^ M; M:L ratio 1:1).

The far-UV-CD spectra of cBDNF(1-12) were obtained by varying the pH values from 4 to 11. The spectra show an intense narrow negative band centered at 199 nm (π → π*) typical of random coil conformation [106] which remains unchanged until pH 8.4 (Figure 1). The contemporary presence of a wide negative band whose intensity increases with the pH values suggests the presence of a small population with a partial structured content, as helix or turn conformation.

The far-UV-CD spectra of cNT3(1-13) run in the 4–11 pH range show an intense narrow negative band centered at 197 nm, whose intensity increases in function of the pH and diagnostic of random coil conformation as found for cBDNF(1-12). The presence of a weak positive band around 227 nm at acidic pH value, suggests the presence of a partial content of a PPII-like conformation [107] and/or a contribute of tyrosines aromatic side chain [108]. Increasing the pH, this band decrease and is blue shifted suggesting a partial content of turn conformation.

The features of far-UV-CD spectra of copper(II) complexes with cBDNF(1-12) do not show appreciable differences respect to those of the free peptide, indicating that copper binding does not affect peptide conformation of cBDNF(1-12). Differently those with cNT3(1-13) show changes increasing the pH, suggesting that copper binding induces modification in the peptide backbone conformation. These data indicate are in agreement with the presence of an extra copper binding site in the primary sequence of cNT3(1-13) that encompasses a second histidine residue, while cBDNF(1-12) contains one histidine metal anchor alone.

### 3.2. cBDNF(1-12) and cNT3(1-13) Show Different Speciation and Binding Affinity for the Copper(II) Ion

The protonation and Cu^2+^ affinity constant values of cBDNF(1-12) and cNT3(1-13) are listed in Table 2 and in Table 3, respectively.

The first protonation constant value of cBDNF(1-12) is attributable to the protonation of the imidazole nitrogen of the His residue and its pK value is in good agreement with those reported in the literature [97,109]. The lowest two protonation constant values can be attributed to the aspartate and glutamate carboxylate side chains and are in accordance with those found for linear peptides that contain the Arg residue [110].

The highest pK values of cNT3(1-13) involve both Tyr and Lys residues; the successive pK values can be attributed to the protonation of the imidazole nitrogen of the two His residues, while the two lowest pK values refer to the two glutamate carboxylate side chains. The amino acid residues take up protons in overlapping steps and thus each protonation constant value should be considered as a macro-constant. The pK values obtained for different protonation centres are similar to those of analogous linear peptides [98,111].

**Figure 2:**
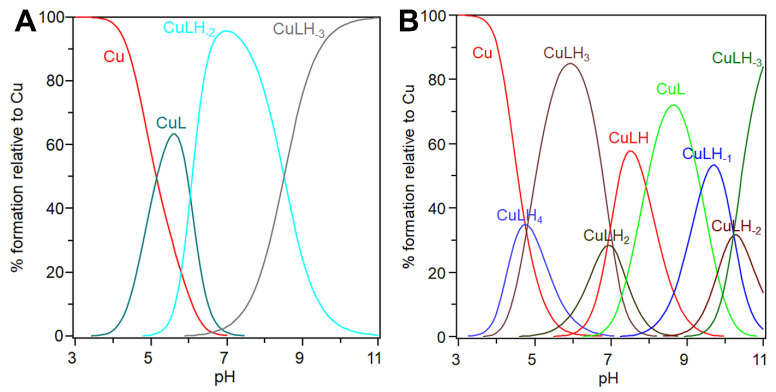
Species distribution of copper(II) complexes with (**A**) cBDNF(1-12) and (**B**) cNT3(1-13). [L] = 1 × 10^−3^ M. M/L molar ratio = 1:1.

Table 3 shows the stability constants determined for the copper(II) complex species with cBDNF(1-12) and cNT3(1-13). Figure 2 shows the distribution diagrams of copper(II) complexes with the cyclic peptides, determined over the pH range 3.0–11.0.

cBDNF(1-12) has three different copper(II) ion binding sites in the pH range investigated: (i) the imidazole of histidine, His-1 and (ii) the carboxylate side chains of aspartic and glutamic acid, Asp-3 and Glu-9. The first metal complex detected at acid pH is [CuL], which is the main species up to pH = 6 (Figure 2A).

Its log β value (4.77) (Table 3), the UV-vis (λ_max_ = 688 nm) and CD parameters (bands at 254 nm and 337 nm representative of N_Im_ π_2_ → Cu(II) charge transfer and N_Im_ → Cu(II) transitions, (Table 4) suggest the involvement of the imidazole nitrogen atom and the deprotonated carboxylate groups in Cu^2+^ coordination. Indeed, the analogous copper(II) complex species formed with the linear BDNF peptide and other cyclic peptides display a lower stability constant value and higher λ_max_ value, according with a 1N1O {N_Im_, O_COO_} coordination environment [97,112].

Increasing the pH value, the [CuLH_−2_] forms due to a cooperative double deprotonation process that promotes the coordination of two amide nitrogen atoms to Cu^2+^. This single-step double deprotonation has been found in similar copper(II)-peptide systems, in which the carboxylate side chain(s) is directly involved in metal binding [113]. The UV–vis spectra deconvoluted for the complex species show a blue-shifted band centered at 590 nm, that suggests a 3N1(2)O {N_Im_, 2N^−^, COO^−^} metal coordination environment, as found already in similar copper(II) complexes with linear peptides [111,114]. CD spectra (see Table 4) calculated for [CuLH_−2_] complex species show a band centered at 254 nm attributable to the N_Im_ π_2_ → Cu(II) charge transfer, a band with a maximum centered at 340 nm in which are present together the N_Im_ → Cu(II) and N_N−_ → Cu(II) charge transfer transitions. Finally, the d-d transition band is centered at 578 nm, characterized by a negative maximum. The log K value of [CuLH_−3_] that starts to form around pH 7 indicates the binding of another nitrogen atom to the metal ion, giving rise to a 4N {N_Im_, 3N^−^} coordination environment [115]. The 4N coordination mode is supported by the blue-shifted UV-vis d-d λ_max_ centered at 546 nm. In the CD spectra, the bands (see Table 4) calculated for [CuLH_−3_] exhibit: (i) the N_Im_ π_2_ → Cu(II) charge transfer band which red-shifted to 270 nm (probably due to a slightly tilted disposition of the imidazole respect to the coordination plane formed by the three nitrogen atoms), (ii) the band centered at 308 nm due to the N_N−_ → Cu(II) charge transfer transitions; (iii) the blue-shift of the band relative to the d-d transition now centered at 555 nm, together with the presence of a second d-d transition band with λ_max_ centered at 479 nm.

cNT3(1-13) has four copper(II) ion binding sites in the pH range investigated: (i) the two carboxylate side chains of the two glutamic acid, Glu-3 and Glu-10, and (ii) the two imidazole of the two histidine, His-4 and His-7. The side chains of tyrosines, Tyr-1 and Tyr-11, and lysine, Lys-5, start deprotonation at strongly basic pH and according to potentiometric data are not directly involved in the metal binding (see Table 2).

The distribution diagram of copper(II) complexes with cNT-3(1-13) shows that [CuLH_4_] forms with an abundance up to 30% at pH 5 and it coexists with free Cu^2+^ and the [CuLH_3_] species (Figure 2B). The stability constant value for this equilibrium step, (log K_114_ = log β_(114)_ − log β_(014)_ = 4.8), suggests the involvement in the copper(II) coordination of a deprotonated N_Im_ and two COO^−^ groups, recalling the coordination environment found for the [CuL] complex species of copper(II)-cBDNF(1-12) system. Since the lysine/tyrosine amino acids are still protonated, the actual species is [CuLH(H_3_)] with one of the histidine imidazole protonated. The spectroscopic characterization of this complex species was hindered due to the extensive overlap of different species spectra (Figure 2B). Increasing the pH value, the [CuLH_3_] predominates up to pH = 6. The stepwise constant (logK_113_ = log β_(113)_ − log β_(114)_ = 4.87) is well-matched with the second histidine imidazole nitrogen binding, thus giving rise to a 2N2O coordination environment of Cu^2+^ in [CuL(H_3_)], in keeping with a similar system [98]. The UV-vis absorption band (λ_max_ = 688 nm; ε = 55 M^−1^ cm^−1^), the CD N_Im_ π_2_ → Cu(II) charge transfer band (λ_max_ = 260 nm; Δε = 1.31 M^−1^ cm^−1^) determined for this complex species, are consistent with the above-mentioned {2N_Im_, 2O_COO_} Cu^2+^ coordination mode [116,117]. On further raising the pH value, [CuLH_2_] forms but its formation percentage is significantly lower than that both of [CuLH_3_] and [CuLH] and for this reason can be characterized by the thermodynamic data alone. The value of the stepwise constant, (log K_112_ = log β_(113)_ − log β_(112)_ = 7.04), suggests the deprotonation of one amide nitrogen atom, giving rise to a 3N1O {2N_im_, 1N^−^, 1O_COO_} chromophore. The actual species is [CuLH_−1_(H_3_)] considering that the tyrosine and lysine residues are still protonated. [CuLH] is the prevalent species at physiological pH and its stepwise constant value (log K_111_ = log β_(112)_ − log β_(111)_ = 6.91) is consistent with a 4N coordination mode, in which a second deprotonated amide nitrogen atom results involved in copper(II) ion coordination. The UV-vis spectrum features (band centered at 595 nm and ε = 65 M^−1^ cm^−1^) recall those found for similar peptide sequences (HXH or HXXH), in which two histidine residues are spaced apart by one or two amino acid residues [118] and support the suggested metal binding details which are corroborated by CD spectra features. In fact, the spectra are characterized by both a band with a λ_max_ at 260 nm, and another with a λ_max_ at 316 nm attributable to the N_Im_ π_2_ → Cu^2+^ and N_N−_ → Cu^2+^ charge transfer transition, respectively. In addition, the N_Im_ → Cu(II) transition band is found at 356 nm, while the d-d transitions in the visible region are characterized by two bands centered at 475 nm and 553 nm, respectively.

Then, a [CuL] complex species forms, reaching its maximum formation percentage before pH 9, at this pH tyrosine and lysine are protonated and the actual species is [CuLH_−3_(H_3_)]. The stepwise constant value (log K_110_ = log β_(111)_ − log β_(110)_ = 7.92) suggests the deprotonation of a further peptide nitrogen atom, leading to the formation of a 4N species. This complex species is characterized by the involvement of three deprotonated amide nitrogen atoms and one imidazole nitrogen atom of a histidine residue, with a copper(II) ion that experiences a {1N_im_, 3N^−^} coordination environment. This metal binding of the peptide is supported by a blue shift of the λ_max_ which moves from 596 nm to 512 nm, and is corroborated by: (i) the intensity increase of the N_N−_ → Cu^2+^ charge transfer transition band centered at 319 nm; (ii) the decrease of the maximum of the N_Im_ → Cu^2+^ transition band centered at 358 nm; (iii) the increase of the intensity of the d-d transition band found now at 612 nm.

Moreover, the stepwise constant values associated with the formation of [CuLH_−1_], [CuLH_−2_], [CuLH_−3_] complex species are comparable to those for the protonation steps of the two Tyr and the Lys residues found for the metal-free ligand. The UV-vis and CD spectra parameters (Table 4) are practically unvaried in comparison with those of the previous species, hence confirming that Tyr and Lys are not involved in the metal binding, but contribute only to the variation of the total charge of the copper(II) complexes with cNT3(1-13).

In summary, the two cyclic peptides show different speciation and binding features in their copper(II) complexes.

### 3.3. Conditional Dissociation Constant Values (^c^K_d_) Allow for a Comparison of Different Affinity of the Two Peptides

Conditional dissociation constants were calculated to compare the ability of cBDNF(1-12) and cNT3(1-13) peptides to complex Cu^2+^; this approach allows an extension of the comparison to the affinity of copper(II) complex with cNGF(1-14), previously investigated [102].

The calculation was carried out at pH = 7.4 by using the method reported in the literature [119].

The dissociation constant associated to equilibrium: ML ⇌ M + L, is given by

^c^K_d_ = [M][L]/[ML].

^c^K_d_ is not the value of the dissociation constant of a metal–biomolecule complex but it rather represents the ‘average’ of the dissociation constants of all the species present in solution regardless of their stoichiometries and structures [120,121,122].

The calculated conditional dissociation constants (^c^K_d_) for the interaction of the three cyclic peptides with Cu^2+^ are listed in Table 5.

The ^c^K_d_ values for cNT3(1-13) and cBDNF(1-12) interaction with Cu^2+^ are comparable to each other, and they appear different from that for cNGF(1-14) due to the distortion of the coordination environment that characterizes the metal binding of the NGF mimicking peptide.

### 3.4. Copper(II) Ion Is Present at Sub-Micromolar Concentration in Culture Media Supplemented with Serum

Most cell types in culture require inorganic trace element bioavailability to grow, proliferate and differentiate, and bio-metals present in aqueous solution and other components of cell culture medium guarantee these fundamental cellular processes. The serum usually added as a supplement at different dilution levels contributes to the metal ion composition of complete cellular medium, [123] while further amount of trace elements can also originate from biometals present in buffers [124]. Despite the increased interest in metallostasis [125,126], the determination of micronutrient concentrations, such as copper, zinc, manganese, cobalt, molybdenum and selenium, present in culture medium and in serum is often overlooked. Moreover, being aware of a study that highlights the fluctuation of copper(II) ion concentrations between different batches of Minimal Essential Medium (MEM) and Dulbecco’s Modified Eagle Media (DMEM) from the same suppliers and between different suppliers [127], ICP-OES was employed to determine the copper content of both the cell culture media and the serum used to supplement the media. The Cu^2+^ amount in the culture medium utilized for cell growth, DMEM/F12, 0.02 μM, rises to 0.25 μM following the addition of 10% FBS, while the Cu^2+^ level in the culture medium employed for peptide treatments, DMEM High Glucose, 0.04 μM rises to 0.05 μM following the addition of 0.5% FBS. Overall, the results suggest that the Cu^2+^ present in the culture media supplemented with serum can affect cBDNF(1-12) and cNT3(1-13) activities before the addition of exogenous CuSO_4_.

### 3.5. cNT3(1-13) and cBDNF(1-12) Mimic the Neuronal Differentiation Capability of NTs in SH-SY5Y Neuroblastoma Cells

Since neurite outgrowth is a marker of neuronal differentiation [100], neurite lengths are assumed as markers of morphological changes associated with neurodifferentiation.

**Figure 3:**
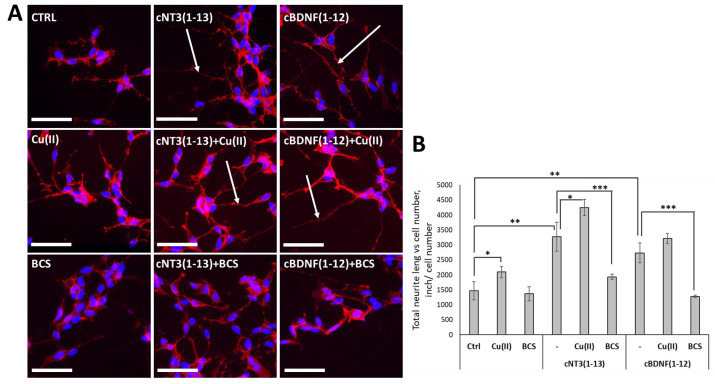
cNT3(1-13) and cBDNF(1-12) induce SH-SY5Y cells’ neuron-like differentiation. SH-SY5Y cells were treated with or without peptides (60 µM), in the presence of CuSO_4_ (10 µM) or BCS (medium pre-treated with BCS 50 µM for 24 h). (**A**) Representative fluorescence microscopy images stained for cell membrane (red, Wheat Germ Agglutinin) in non-permeabilized SH-SY5Y cells treated for 72 h. Cell nuclei were counterstained with Hoechst 33342 (blue). (**B**) Quantitative analysis of neurite length after 72 h performed with Image J. Data are expressed as mean ± SD of four images per well acquired at each time point during two experiments conducted in triplicate. * *p* ≤ 0.05, ** *p* ≤ 0.01, *** *p* ≤ 0.001 as a result of the one-way ANOVA followed by Tukey’s test. Data concerning each single treatment and untreated cells, within either the cNT3(1-13) or the cBDNF(1-12) experiment, were compared as indicated by the brackets for each specific comparison. Scale bar, 66 μm. Magnification, 40×.

Specifically, copper treatment induces neuron-like phenotype by stimulating neurite growth (2095 ± 184 Inch/cell number) compared to control cells (1471 ± 304 Inch/cell number), while BCS did not affect the neurite length (1368 ± 236 Inch/cell) compared to control (Figure 3A,B), demonstrating the importance of copper content in cell culture medium in stimulating neuro-differentiation.

Both peptides cNT3(1-13) and cBDNF(1-12) exert no toxic effect on SH-SY5Y cells and promote neuronal differentiation by inducing neurite outgrowth, clearly visible after 72 h (3271 ± 485 and 2733± 329 Inch/cell number for cNT3(1-13) and cBDNF(1-12), respectively) (Figure 3A,B). Copper treatment in combination with cyclic peptides significantly enhances neuronal differentiation of neuroblastoma cells (4245 ± 272 Inch/cell number for cNT3(1-13) and 3215 ± 167 Inch/cell number for cBDNF(1-12)). Conversely, BCS treatment markedly reduces the peptides-induced neurite growth (1930 ± 91 and 1278 ± 45 Inch/cell number for cNT3(1-13) and cBDNF(1-12), respectively) (Figure 3A,B).

The induction of neurogenesis is one of the effects of neurotrophins, overall the cyclic peptides concur with Cu^2+^ to neuronal differentiation.

### 3.6. cNT-3(1-13) and cBDNF(1-12) Are Ionophore Peptides and Induce a Decrease of Ctr1 Localization on Cell Membrane

**Figure 4:**
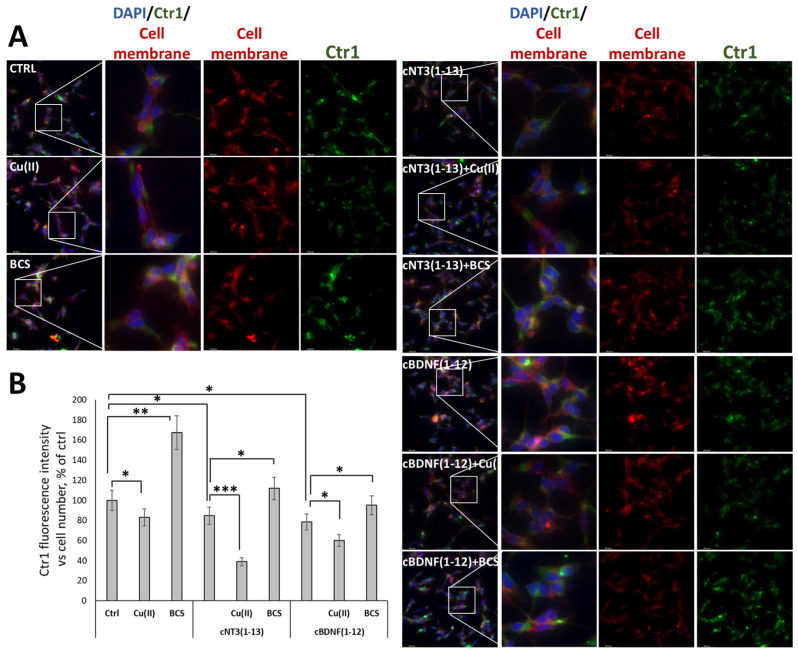
cNT3(1-13) and cBDNF(1-12) affect intracellular copper homeostasis through CTR1 membrane localization. SH-SY5Y cells were treated with or without peptides (60 µM), in the presence of CuSO_4_ (10 µM) or BCS (medium pre-treated with BCS 50 µM for 24 h). (**A**) Representative immunofluorescence images stained for cell membrane (red), extracellular domain of Ctr1 (green) in non-permeabilized SH-SY5Y cells treated for 72 h and (**B**) quantification of Ctr1 fluorescence intensity expressed as % of untreated control cells. Data are expressed as mean ± SD of four images per well acquired at each time point during two experiments conducted in triplicate. * *p* ≤ 0.05, ** *p* ≤ 0.01, *** *p* ≤ 0.001 as a result of the one-way ANOVA followed by Tukey’s test.Cell nuclei were counterstained with Hoechst 33342 (blue). Data concerning each single treatment and untreated cells, within either the cNT3(1-13) or the cBDNF(1-12) experiment, were compared as indicated by the brackets for each specific comparison. Scale bar, 66 μm. Magnification, 40×.

The membrane localization and the cytoplasmic translocation of high-affinity copper importer Ctr1 in SH-SY5Y cells exposed to peptides are investigated by immunofluorescence staining. As expected, the treatment with 10 µM CuSO_4_ decreases copper transporter levels on the cell surface (83 ± 9%) while BCS with its metal chelating properties causes an increase in Ctr1 (167 ± 13%) compared to control cells (Figure 4A,B).

After 72 h of peptide stimulation, Ctr1 level on the cellular membrane decreases (85 ± 9% for cNT3(1-13) and 77 ± 7% for cBDNF(1-12)), similarly to what is observed for copper treatment alone (83 ± 9%), suggesting that the peptide as ionophore favors metal influx of the copper present in the culture medium. Incubation with cNT3(1-13) or cBDNF(1-12) in the presence of CuSO_4_ decreases the membrane Ctr1 level (39 ± 4% for cNT3(1-13) and 60 ± 7% for cBDNF(1-12)). Consistently, BCS addition to the peptides treatment induces a significant Ctr1 turning back onto the cell surface (112 ± 15% for cNT3(1-13) + BCS and 95 ± 10% for cBDNF(1-12) + BCS). Therefore, the peptides promote translocation of Ctr1 from the membrane to the cytoplasm as a result of increased intracellular copper intake. Overall, during cyclic peptide-dependent neuroblastoma differentiation, we can state that changes in copper homeostasis and localization of the main membrane copper transporter occur.

### 3.7. cNT3(1-13) and cBDNF(1-12) Increase TrkA and TrkB as Well as VEGFR1 and VEGFR2 Receptors Expression

Both BDNF and NT-3 proteins display not only their trophic activity but also act as angiogenic factors as found in a mouse model of limb ischemia [128].

**Figure 5:**
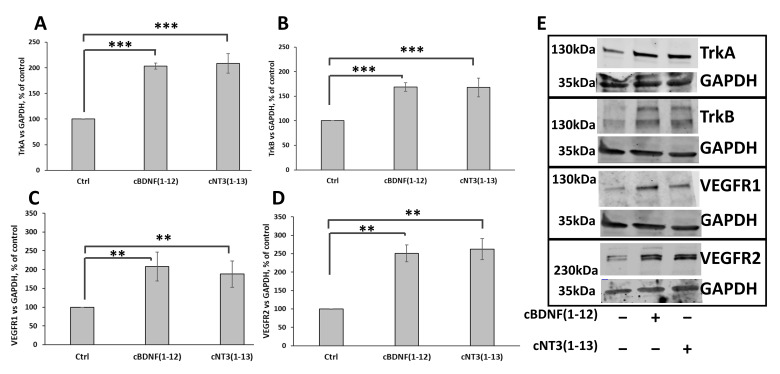
cNT3(1-13) and cBDNF(1-12) modulate TrkA, TrkB, VEGFR1 and VEGFR2 expression. SH-SY5Y cells were treated with or without cNT3(1-13) (60 µM) and cBDNF(1-12) (60 µM) for 72 h. (**A**–**D**) Densitometric analysis and (**E**) representative western blotting images of TrkA (**A**), TrkB (**B**), VEGFR1 (**C**) and VEGFR2 (**D**) in SH-SY5Y cells. The expression level of TrkA, TrkB, VEGFR1 and VEGFR2 is normalized to GAPDH and expressed as a percentage of control cells. Data are expressed as mean ± SD. ** *p* ≤ 0.01, *** *p* ≤ 0.001 as a result of the one-way ANOVA followed by Tukey’s test. Data concerning each single treatment and untreated cells, within either the cNT3(1-13) or the cBDNF(1-12) experiment, were compared as indicated by the brackets for each specific comparison.

To verify the ability of the two cyclic peptides to mimic this dual capacity of the proteins, the influence of cNT3(1-13) and cBDNF(1-12) on the expression of the receptor tyrosine kinases, TrkA and TrkB, and VEGF receptors 1 and 2 was investigated. The expression of these receptors results strongly enhanced by the two cyclic peptides (TrkA, 203 ± 6% for cBDNF(1-12) and 208 ± 19% for cNT3(1-13); TrkB, 169 ± 15% for cBDNF(1-12) and 168 ± 40% for cNT3(1-13); VEGFR1, 208 ± 38% for cBDNF(1-12) and 188 ± 34% for cNT3(1-13); VEGFR2, 250 ± 23% for cBDNF(1-12) and 262 ± 28% for cNT3(1-13)) compared to untreated control (Figure 5).

In summary, both cyclic peptides affect the couple of Trks and VEGFRs, but differently within the two families of trophic and angiogenic receptors, stimulating more the expression of TrkA and VEGFR2.

### 3.8. cNT3(1-13) and cBDNF(1-12) Promote Trk Signaling Cascade by CREB Phosphorylation in a Copper Dependent Manner

The neurotrophins family promotes neuronal survival, differentiation, and synaptic function through the signaling of receptor tropomyosin-related kinase (TrKs) [129]. The potential activation of the signaling pathway by cyclic peptides and the role of copper are tested on the phosphorylation cascade. 

**Figure 6:**
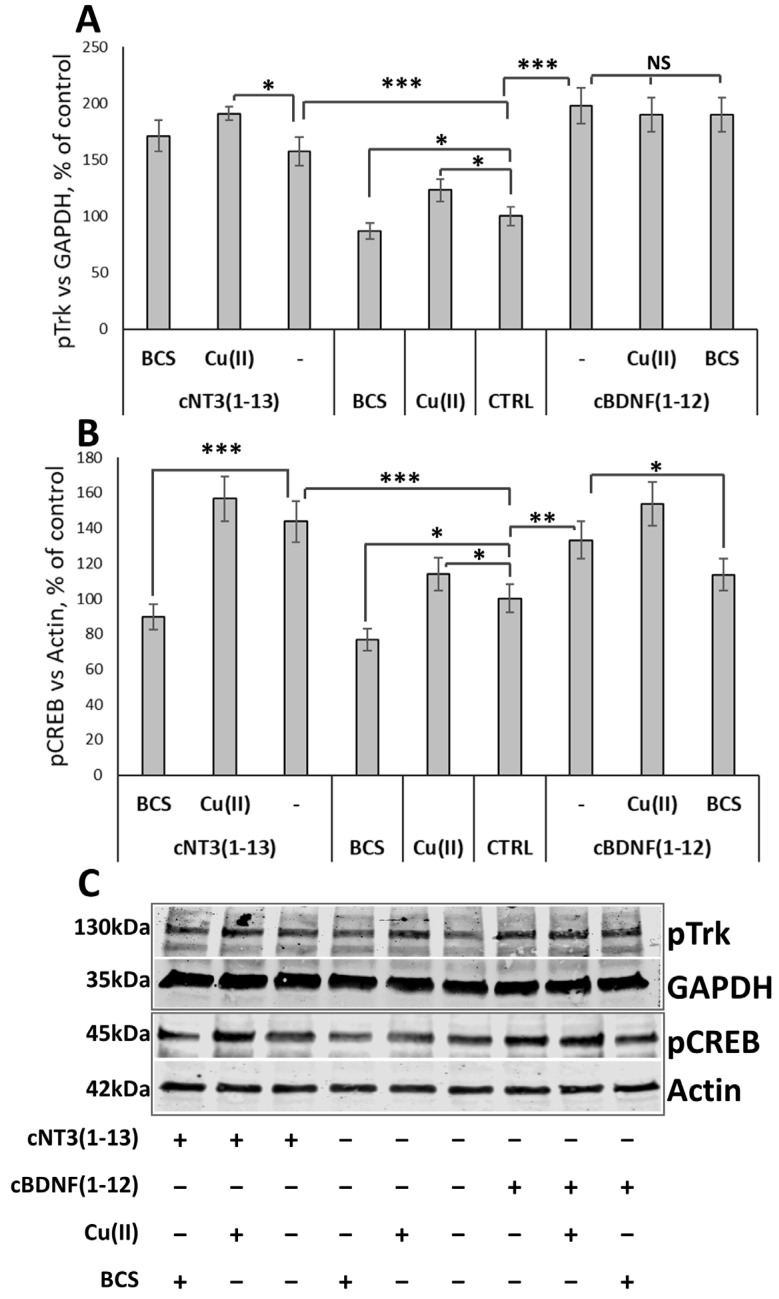
cNT3(1-13) and cBDNF(1-12) activate the Trk—CREB transduction signaling by phosphorylation. SH-SY5Y cells were treated with or without cNT3(1-13) (60 µM) and cBDNF(1-12) (60 µM), in the presence of CuSO_4_ (10 µM) or BCS (medium pre-treated with BCS 50 µM for 24 h). (**A**,**B**) Densitometric analysis and (**C**) representative western blotting image of pTrk, and pCREB in SH-SY5Y cells treated for 10 min and 30 min, respectively. The phosphorylated level of Trk and CREB is normalized to GAPDH or actin and expressed as a percentage of control cells. Data are expressed as mean ± SD. NS *p* > 0.05, * *p* ≤ 0.05, ** *p* ≤ 0.01, *** *p* ≤ 0.001 as a result of the one-way ANOVA followed by Tukey’s test. Data concerning each single treatment and untreated cells, within either the cNT3(1-13) or the cBDNF(1-12) experiment, were compared as indicated by the parentheses for each specific comparison.

Copper treatment increases the cellular level of Trk phosphorylation (123 ± 9%) after 10 min, whereas BCS does not significantly affect Trk activation with respect to control cells. After cNT3(1-13) and cBDNF(1-12) exposure, the Trk phosphorylation is significantly increased by peptides, 158 ± 13% for cNT3(1-13) and 198 ± 16% for cBDNF(1-12) versus untreated controls. Concomitant treatment with copper induces a significant increase in Trk phosphorylation with cNT3(1-13) (196 ± 6%). (Figure 6A,C).

Conversely, after 30 min the analysis of pCREB reveals that copper is strongly involved in this signal transduction. In fact, copper treatment increases CREB phosphorylation while BCS addition drastically reduces it (76 ± 6%). As well as for pTrk, cyclic peptides enhance the levels of pCREB (144 ± 11% for cNT3(1-13); 133 ± 10% for cBDNF(1-12)). In the presence of BCS this effect is reverted (89 ± 7% for cNT3(1-13); 113 ± 9% cBDNF(1-12)) (Figure 6B,C).

### 3.9. cNT3(1-13)/ and cBDNF(1-12)/Trk Pathway Stimulate Expression of Trophic Factors and the Release of VEGF

**Figure 7:**
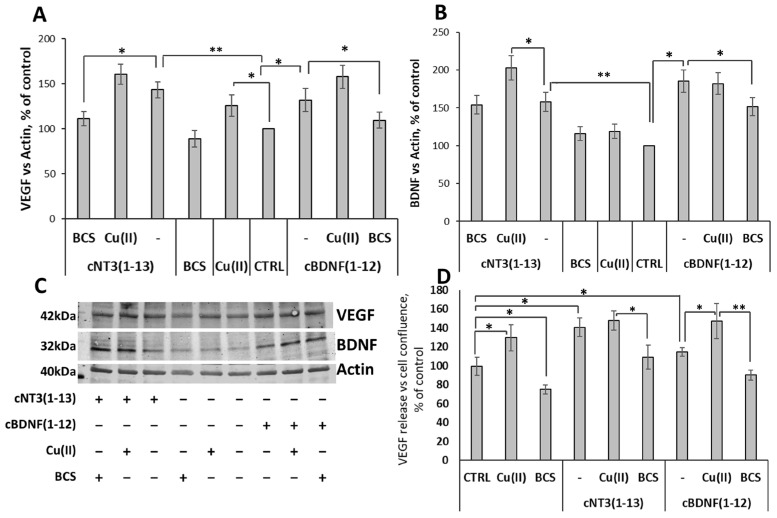
cNT3(1-13) and cBDNF(1-12) stimulate the production of trophic factors BDNF and VEGF. SH-SY5Y cells were treated with or without cNT3(1-13) (60 µM) and cBDNF(1-12) (60 µM), in the presence of CuSO_4_ (10 µM) or BCS (medium pre-treated with BCS 50 µM for 24 h). (**A**,**B**) Densitometric analysis and (**C**) representative western blotting image of VEGF (**A**) and BDNF (**B**) in SH-SY5Y cells treated for 24 h. The VEGF and BDNF expression levels are normalized to actin and expressed as a percentage of control cells. (**D**) VEGF release from SH-SY5Y cells treated with or without peptides (60 µM), in the presence of CuSO_4_ (10 µM) or BCS (medium pre-treated with BCS 50 µM for 24 h) for 24 h. Data are expressed as mean ± SD. * *p* ≤ 0.05, ** *p* ≤ 0.01 as a result of the one-way ANOVA followed by Tukey’s test. Data concerning each single treatment and untreated cells, within either the cNT3(1-13) or the cBDNF(1-12) experiment, were compared as indicated by the brackets for each specific comparison.

To evaluate the role in neurogenesis of VEGF and BDNF induced by cyclic peptides, the release and the expression of both trophic factors are investigated. The treatment of SH-SY5Y cells with copper stimulates VEGF expression (126 ± 12%), whereas BCS does not significantly affect both VEGF and BDNF expression levels compared to control untreated cells. Both peptides cNT3(1-13) and cBDNF(1-12) induce an increase of VEGF expression (144 ± 9% for cNT3(1-13) and 132 ± 13% for cBDNF(1-12), respectively). Analysis of BDNF expression after the treatment only with the peptides significantly increases its level up to 158 ± 13% for cNT3(1-13) and 185 ± 15% for cBDNF(1-12), respectively. The addition of copper slightly enhances the level of VEGF expression up to 161 ± 11% for cNT3(1-13 and 158 ± 13% for cBDNF(1-12), respectively. Furthermore, BDNF expression is affected in cNT3(1-13) cells co-treated with copper (203 ± 16%) (Figure 7A–C). cNT3(1-13) treatment of the cells in the presence of BCS markedly reduces the expression of VEGF until to 111 ± 8%. Incubation with cBDNF(1-12) in the presence of BCS decreases VEGF and BDNF expression (109 ± 9% and 152 ± 12%, respectively).

The release of VEGF enhances after 24 h exposure both to cNT3(1-13) (141 ± 10%) and cBDNF(1-12) (115 ± 4). VEGF release results affected by the additions of copper ions both for cNT3(1-13) up to 148 ± 10 and for cBDNF(1-12) up to 147 ± 19%. However, the involvement of Cu^2+^ is further demonstrated by the effect of BCS addition that decreases the extracellular amount of VEGF both alone (75 ± 5%) and in the presence of both cBDNF(1-12) (90 ± 5%) and cNT3(1-13) (109 ± 13%) (Figure 7D).

All these results clearly highlight that cyclic peptides are able to activate BDNF and VEGF supporting the crosstalk between these neurotrophic molecules as well as the major role of copper in promoting their production.

## 4. Discussion

The multifactorial nature of AD includes the alteration of neurotrophic factors with a decrease in the levels of NT and their related Trks, which concur with the deficit in neurogenesis and synaptic plasticity, associated with neurofibrillary tau and amyloid β oligomers. Taken together, our findings indicate that cBDNF(1-12) and cNT3(1-13) show NT-like activities playing the dual role of trophic and angiogenic factors and promoting CREB phosphorylation together with BDNF and VEGF release.

Peptide mimics of NTs, usually derived from the amino acid sequence of the growth factor itself, show different advantages over other mimics [130], inclusive of lower immunogenicity, enhanced pharmacokinetics and easier handling than proteins, accompanied with the potential for enhanced binding with receptors in comparison with small molecules [131].

To struggle against the inadequate neurotrophic support that features in AD brains [132,133], NT peptide mimetics could represent very promising small molecules to enhance neurogenesis and promote neuronal plasticity, restraining AD cognitive decline [134,135]. As a recently published report highlights [136], neurotrophic peptide mimetics should: (i) cross the Blood Brain Barrier, (ii) undergo a delayed and weak degradation by peptidases [102], and (iii) recognize the same receptor as the NT from which they are originated [137].

Cyclic natural peptides have been recently proposed as promising therapeutic molecular entities, conjugating the advantages of small molecules, such as membrane permeability and oral availability, with the potential ability to interact with protein interfaces.

Different reports put in evidence that BDNF and NT3 [138,139,140] exhibit a significant therapeutic potential in the treatment of neurodegenerative diseases [141,142,143].

Human NT-3 shares 56% of the amino acid identity with human BDNF [144] and this percentage is more or less retained in our cyclic peptides. Far-UV CD spectra (Figure 1) indicate that cBDNF(1-12) and cNT3(1-13) conformation is pH dependent, due to the different deprotonated species which form when increasing the pH; cNT3(1-13) conformation at physiological pH recalls that found for the analogous cNGF(1-14) [102]. Two histidine residues are present in both the peptides and seem to drive their secondary structures, though in solution structural characterization of cyclic peptides through experimental methods is challenging [145]. The histidine residues are the main anchor site for Cu^2+^ binding and the presence of a different number of their imidazole nitrogen donor atoms affects the speciation (Figure 2) and the affinity (Table 3) of the copper(II) complexes with the two cyclic peptides. The second His residue present in cNT3(1-13) delays the formation of its metal complexes with the deprotonated ligand, and different species form in the physiological pH range, while CuLH_−2_ is the lone complex species that cBDNF(1-12) forms in the same pH range. The ^c^K_d_ [120] values could account for these differences between the two cyclic peptides and those related to the previous investigated cNGF(1-14) [102] (Table 5).

The stability constant values and the Cu^2+^ amount determined by ICP-OES allows us to take into account the formation of copper(II) complex species with the peptides employed in the cell treatments, before the addition of 10 μM of copper(II) ions. The simulation findings indicate that the effects attributed to the cyclic peptides alone include a contribution due to all complex species of Cu^2+^ bound to cBDNF(1-12) or cNT3(1-13) that range to around 15% of the total peptide amount. This contribution significantly decreases with the addition of the extracellular chelating agent, BCS, as found for the different cell treatments by the two cyclic peptides, highlighting the role played by the metal ion.

The brain needs significant amount of copper for its functions, including neurotransmitter synthesis, memory and learning processes, myelination, synaptic plasticity and radical scavenging. Under physiological conditions, copper can cycle between the extracellular Cu^2+^ and intracellular Cu^+^ forms. Alteration of copper homeostasis induces apoptosis or cuproptosis in neuronal cells [146,147], while neuronal loss involves memory deficits and cognitive decline in several neurodegenerative diseases [148]. In AD brains the metallostasis [125] perturbation involves Aβ and Tau which are metal proteins and copper binding can promote Aβ oligomerization, which intensifies ROS production and induces oxidative neuronal damage. However, in an attempt to avoid the toxicity of copper overload, in most cells, including brain tissue, cellular copper homeostasis [149] is controlled through copper uptake, which is mediated primarily by the high affinity Cu influx transporter CTR1, which belongs to the solute carrier protein family (SLC31A1) [150]. Otherwise, there are copper chaperones which control the intracellular trafficking and delivery of cytosolic and mitochondrial Cu to their subcellular compartments and final acceptors [151]. Cu efflux can also be mediated by Cu-transporting *p*-type ATPases, ATP7A and ATP7B usually present in the trans Golgi network [152] which export the ion to the ECM and blood, bound to secreted cuproproteins and soluble carriers such as CP and albumin [153]. Metallothioneins (MT1-4) serve as a copper store, regulating its availability [154]. Finally, transcription factors like MTF1 and nuclear ATOX1 control the expression of these Cu^+^ transporters and chaperones to ensure that cells maintain their fundamental Cu needs [155,156].

In addition to this complex network which regulates metallostasis [125], different classes of chelating molecules with ionophore abilities are reported to restore metal homeostasis in AD cellular models, in preclinical animal studies as well as in human clinical trials [157]. These metal ligands are able to compete with the toxic binding of Aβ with the metal ion outside the cell and relocate it into intracellular compartments, modulating and counteracting AD progression [158,159,160,161]. All these ligands share the capacity to activate the kinase cascade pathways and upregulate matrix metalloproteases, inducing Aβ degradation [159].

Recently, our knowledge of the modulation of metallostasis [125] in brain has increased [149,162,163]; consistently, and the essential role played by Ctr1 in metal transfer into the brain is emerging together with its involvement in new neurological disorders [164]. cBDNF(1-12) and cNT3(1-13) affect Ctr1 localization on the cell membrane, inducing its endocytic process that indicates that the copper cellular importer senses the metal transfer inside the cell due to the ionophore ability of the NT mimics. This effect attributable to the two peptide binding to Cu^2+^ present in the culture medium increases by adding an exogenous metal ion (10 μM), while BCS counteracts this process in a way roughly dependent on the different copper(II) ion affinities of the two cyclic peptides (Figure 4).

Different NTs can contribute to favoring neurogenesis [165] which is decreased in AD brains [48]. In animal models of AD, genetic manipulations can induce a blockade of neurogenesis that is associated with worsening cognitive deficits. On the other hand, stimulation of neurogenesis in in vivo AD models increases memory processes [166,167]. At a molecular level, APP itself affects neurogenesis, so that abnormalities in this Aβ progenitor can induce an alteration in this relevant process for healthy brains [168]. These evidences suggested that adult neurogenesis has a critical role in AD pathogenesis [169,170,171].

Searching for NT mimetics involves finding of active compounds in the key physiological functions of NTs, namely, differentiation (neurogenesis), development (neurite outgrowth promotion), and survival (protection from neuronal death) of neurons. Our peptides mimicking the NT features are able to promote SH-SY5Y cells’ neuron-like differentiation and neurite outgrowth with associated neurogenesis (Figure 3). These effects are increased by adding metal ions, recalling what was already reported about copper induced neuronal differentiation [172] and its ability to modulate adult neurogenesis [173,174].

TrkA, TrkB, and TrkC share similar structural arrangements [175] that encompasses five extracellular domains (domains 1 and 3 are cysteine-rich regions, domain 2 a leucine-rich region, and domains 4 and 5 are immunoglobulin-like domains), a transmembrane region, and the intracellular kinase domain. TrkB and TrkC findings indicate that domain 5 (D5) is sufficient for the binding of NTs and is responsible for their binding specificity. The kinase domains of TrkA, TrkB, and TrkC share between 71.9% and 78.3% sequence identity, TrkB and TrkC being the closest homologues [176]. Trk kinases possess an additional less common structural element: the kinase insert domain (KID). The KID can be found in the VEGFR protein kinase family and consists of an extension of the loop located between helices α-D and α-E of the C-terminal lobe. Studies on deletion or mutations of the KID domain on kinases of the VEGFR family showed that the KID is not important for the intrinsic kinase activity, but it is important for the binding of other proteins involved in signal transduction via auto-phosphorylation of KID tyrosine residues [177]. The NTs/Trks system is essential for the process of both embryonic and adult neurogenesis, and its signaling alteration is involved in impaired neurogenesis during aging and/or in the pathogenesis of brain diseases including AD [178,179]. cBDNF(1-12) and cNT3(1-13) imitate their related proteins and induce Trk phosphorylation in a way that appears enhanced by copper (Figure 6). This is consistent with the findings that Cu^2+^ stimulates the Trk-mediated signal pathways in a ligand-independent mode probably driving the dimerization of Trk monomers [180]. This suggested mechanism appears reasonable, recalling the role played by the histidine residues present in the N-terminus of NGF for its binding to the TrkA-D5 domain [181] and the recognized anchor role of histidine imidazole nitrogen atoms for copper binding as reported here for the two cyclic peptides (Table 3 and Table 4). Recently, computational and experimental findings have supported this mechanism, based on utilizing NT mimetics encompassing histidine residues in the copper-assisted activation of Trk phosphorylation by cNGF(1-14) [102], in agreement with the analogous linear peptides [96,99].

Trks phosphorylation activates CREB whose signaling pathways are impaired in AD brain patients [182], thus exacerbating synaptic dysfunction and memory loss [183]. A recent study reports that copper blocks CREB phosphorylation, thereby reducing the expression of its downstream target protein, brain-derived neurotrophic factor (BDNF), leading to cognitive dysfunction in mice [147]. The copper concentration (80 μM) employed in the biological assays is a clear example of copper overload that is significantly higher than that present in the culture medium (sub-micromolar) and in the added copper solution (10 μM) employed in our assays. The cyclic peptides promote CREB phosphorylation mimicking their related proteins and this effect is amplified by copper addition (Figure 6). cBDNF(1-12) and cNT3(1-3) triggers CREB transcription activity, inducing the production of BDNF and VEGF with the contribution of Cu^2+^ that also concurs with VEGF release (Figure 7).

Trks and VEGFRs are downregulated in AD [184,185]. In different contexts, NT treatments [186,187] and voluntary exercises, which promote BDNF expression [188,189] or addition of different molecules [190] are demonstrated to induce the expression of these tyrosine kinase receptors. Interesting, cBDNF(1-12) and cNT3(1-13) are able to mimic these features of their related proteins and induce the expression of Trks and VEGFRs (Figure 5); to the best of our knowledge it is the first time that NT mimetics have shown these abilities that can promote neurogenesis and angiogenesis in AD.

## Figures and Tables

**Table 1:** List of primary antibodies used and experimental applications.

Protein	Company and Code	Dilution and Application
phospho-Trk	SantaCruz (Santa Cruz, CA, USA), sc-8058	1:500 for western blotting assay
phospho-CREB	SantaCruz (Santa Cruz, CA, USA), sc-8058	1:500 for western blotting assay
VEGFR1	SantaCruz (Santa Cruz, CA, USA), sc-271789	1:500 for western blotting assay
VEGFR2	SantaCruz (Santa Cruz, CA, USA), sc-6251	1:500 for western blotting assay
TrkA	Cell Signaling (Danvers, MA, USA), #2505	1:800 for western blotting assay
TrkB	Abcam Waltham, MA, USA), ab33655	1:1000 for western blotting assay
anti-Ctr1	Abcam Waltham, MA, USA), ab129067	1:3000 for western blotting assay 2.5 µg/mL for immunofluorescence assay
anti-VEGF	SantaCruz, sc-72693	1:500 for western blotting assay 1:50 for ELISA
anti-BDNF	SantaCruz, sc-65513	1:500 for western blotting assay
anti-GAPDH	Abcam (Waltham, MA, USA), ab8245	1:3000 for western blotting assay
anti-Actin	Sigma Aldrich (St. Louis, MO, USA), A3853	1:2000 for western blotting assay

**Table 2:** Protonation constants (log β ^a^ and pK ^b^ values) for cBDNF(1-12) and cNT3(1-13) (T = 298 K, I = 0.1 M, KNO_3_).

Species [LqHr] ^c^	cBDNF(1-12)		cNT3(1-13)	
	log β ^d^	pK	log β ^d^	pK
11	6.60 (3)	6.60	-	-
12	11.31 (4)	4.71	20.01 (1)	10.00 × 2
13	15.07 (4)	3.76	29.11 (2)	9.10
14	-	-	35.78 (3)	6.67
15	-	-	41.81 (3)	6.03
16	-	-	46.36 (4)	4.55
17	-	-	50.31 (3)	3.95

^a^ Overall stability constants (β) expressed by the equation: β (L_q_H_r_) = [L_q_H_r_]/([L]^q^[H]^r^); ^b^ Acid dissociation constants (p*K*) expressed by: p*K* = log β (LH_r_) − log β (LH_r−1_). ^c^ Charges are omitted for simplicity. ^d^ Standard deviations (3σ values) in parentheses.

**Table 3:** Stability constants (log β ^a^ and pK ^b^ values) for Cu^2+^ complexes with cBDNF(1-12) and cNT3(1-13). (T = 298; I = 0.1 M KNO_3_).

Species [Cu_p_L_q_H_r_] ^c^	cBDNF(1-12)		cNT3(1-13)	
	log β ^d^	pK	log β ^d^	pK
114	-	-	40.58 (1)	-
113	-	-	35.77 (1)	4.81
112	-	-	28.73 (3)	7.04
111	-	-	21.82 (1)	6.91
110	4.77 (5)	-	13.90 (2)	7.92
11-1	-		4.57 (3)	9.34
11-2	−7.30 (4)	6.03 × 2	−5.68 (2)	10.24
11-3	−15.81 (5)	8.51	−15.88 (2)	10.20

^a^ pCu + qL + rH ⇆ Cu_p_L_q_H_r_; β_pqr_ = [Cu_p_L_q_H_r_]/[Cu]^p^[L]^q^[H]^r^. ^b^ pK(n/m) values refer to the pK values of Cu^2+^ complexes. ^c^ Charges are omitted for simplicity. ^d^ Standard deviations (3σ values) are given in parentheses.

**Table 4:** Spectroscopic parameters of copper(II) complexes with cBDNF(1-12) and cNT3(1-13).

L	Species [Cu_p_L_q_H_r_]	UV-Vis λ (nm) (ε, M^−1^ cm^−1^)	CD λ (nm) (Δε, M^−1^ cm^−1^)
	CuL	686 (75)	254 (+1.11); 337 (+0.13)
cBDNF(1-12)	CuLH_−2_	590 (99)	254 (+3.17); 340 (+1.33); 578 (−0.78)
	CuLH_−3_	546 (117)	270 (+3.54), 308 (+1.51), 479 (+0.38), 555 (−1.31)
	CuLH_3_	688 (55)	260 (+1.31)
	CuLH	596 (65)	260 (+2.01), 316 (+0.15), 356 (−0.20), 475 (−0.19), 553 (+0.10)
cNT3(1-13)	CuL	512 (113)	260 (+4.22), 319 (+0.90), 358 (−0.15), 488 (−1.07), 612 (+0.81)
	CuLH_−1_	512 (120)	260 (+4.50), 319 (+0.95), 358 (−0.12), 490 (−1.13), 614 (+0.86)
	CuLH_−3_	515 (95)	260 (+4.60), 319 (+0.92), 358 (−0.12), 496 (−0.94), 617 (+0.54)

[L] = 1 × 10^−3^ mol dm^−3^. Errors in λ = ±2 nm and ε = 5%. Charges are omitted for simplicity.

**Table 5:** ^c^K_d_ values of copper(II) complex species with cNT3(1-13), cBDNF(1-12) and cNGF(1-14) at pH 7.4.

Peptide	^c^K_d_ (M^−1^)
cNT3(1-13)	1.99 × 10^−8^
cBDNF(1-12)	3.42 × 10^−8^
cNGF(1-14)	1.49 × 10^−7^

## Data Availability

All data generated or analyzed during this study are included in this published article.

## Supplementary Materials

The following supporting information can be downloaded at: https://www.mdpi.com/article/10.3390/biom14091104/s1, Figure S1: ^1^H-NMR spectrum and the corresponding g-cosy NMR of a sample of cBDNF (1-12). Figure S2: ^1^H-NMR spectrum and the corresponding g-cosy NMR of a sample of cNT3 (1-13).
