# Supplementary material for: New BDNF and NT-3 Cyclic Mimetics Concur with Copper to Activate Trophic Signaling Pathways as Potential Molecular Entities to Protect Old Brains from Neurodegeneration
Source: Biomolecules. 2024 Sep 2;14(9):1104. doi: 10.3390/biom14091104 (PMC11430436; doi:10.3390/biom14091104)
Supplement: Supplementary file 1 [file biomolecules-14-01104-s001.zip › biomolecules-3168643-Updated Supplementary.pdf]

Supplemental Materials

New BDNF and NT-3 Cyclic Mimetics Concur with Copper to Activate Trophic Signaling Pathways as Potential Molecular Entities to Protect Old Brains from Neurodegeneration

Antonio Magrì [1,†], Barbara Tomasello [2,†], Irina Naletova [1,†], Giovanni Tabbì [1,†], Warren R.L. Cairns [3], Valentina Greco [4], Sebastiano Sciuto [4], Diego La Mendola [5] and Enrico Rizzarelli [1,4,*]

[1] Institute of Crystallography, National Council of Research (CNR), P. Gaifami 18, 95126 Catania, Italy; antonio.magri@cnr.it (A.M.); irina.naletova@ic.cnr.it (I.N.); giovanni.tabbi@ cnr.it (G.T.)

[2] Department of Drug and Health Sciences, University of Catania, Viale Andrea Doria 6, 95125 Catania, Italy; btomase@unict.it (B.T)

[3] CNR-Institute of Polar Sciences (CNR-ISP), 155 Via Torino, 30172 Venice, Italy; warrenraymondlee.cairns@cnr.it (W.R.L.C)

[4] Department of Chemical Sciences, University of Catania, Viale Andrea Doria 6, 95125 Catania, Italy; vgreco@unict.it (V.G.); ssciuto@unict.it (S.S.); erizzarelli@unict.it (E.R.)

[5] Department of Pharmacy, University of Pisa, via Bonanno Pisano 6, 56126 Pisa, Italy; diego.lamendola@unipi.it (D.L.M)

* Correspondence: erizzarelli@unict.it

† These authors contributed equally to this work.

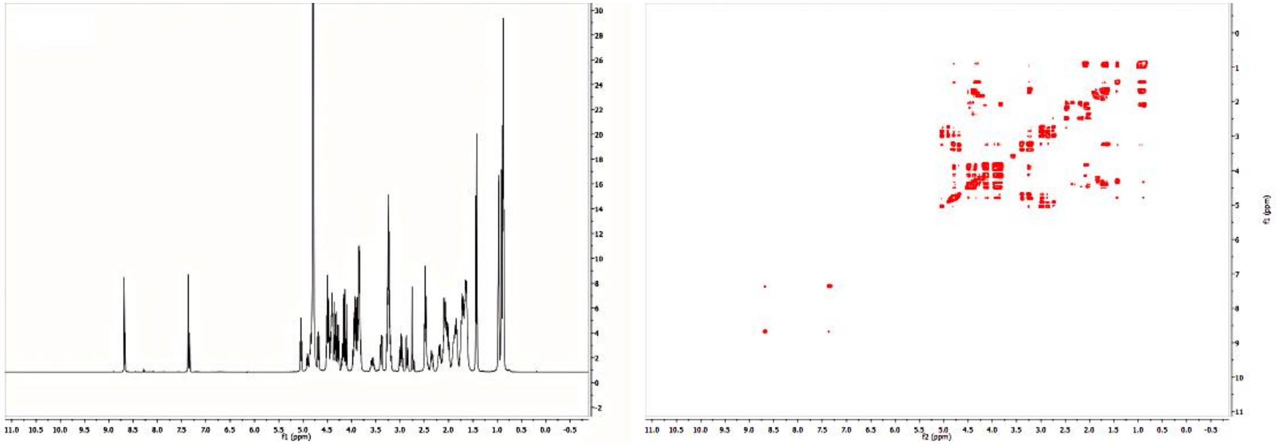

Figure S1 – ^{1}H-NMR spectrum (at the left side, 500 MHz, D_2O, 3 mg/ml) and the corresponding g-cosy NMR Spectrum (at the right side) of a sample of cBDNF(1-12).

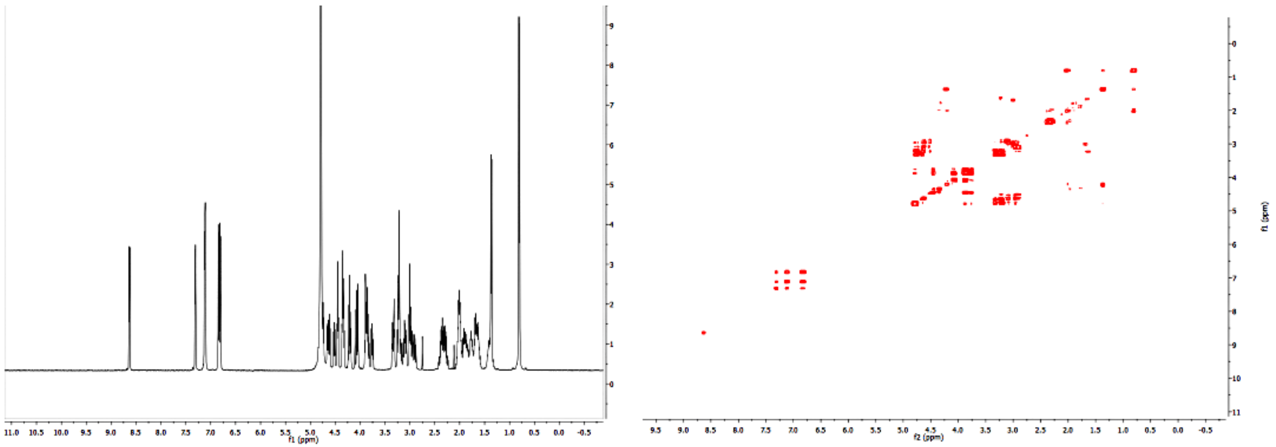

Figure S2– ^{1}H-NMR spectrum (at the left side, 500 MHz, D_2O, 3 mg/ml) and the corresponding g-cosy NMR spectrum (at the right side) of a sample of cNT3(1-13).

Original images of Western Blots

Protein Ladder and the list of antibodies used for western blot detection.

**Protein Ladder**	**Company and Code**	**Volume per well**
Prestained Protein Ladder IRIS9 Plus	BIO-HELIX, code: PMI09-0500	2-5 μL
Precision Plus Protein Standarts Kaleidoscope	BIO-RAD, #1610375	10 μL
**Secondary antibody**	**Company and Code**	**Dilution**
Goat anti-rabbit labeled with IRDye 680	Cat# 926-68071	1:20000
Goat anti-rabbit labeled with IRDye 800	Cat# 925-32211	1:20000
Goat anti-mouse labeled with IRDye 680	Cat# 926-68070	1:20000
Goat anti-mouse labeled with IRDye 800	Cat# 925-32210	1:20000
**Protein**	**Company and Code**	**Dilution**
phospho-Trk	SantaCruz, sc-8058	1:500
phospho-CREB	SantaCruz, sc-81486	1:500
VEGFR1	SantaCruz, sc-271789	1:500
VEGFR2	SantaCruz, sc-6251	1:500
TrkA	Cell Signaling, #2505	1:800
TrkB	Abcam, ab33655	1:1000
anti-Ctr1	Abcam, ab129067	1:3000
anti-VEGF	SantaCruz, sc-7269	1:500
anti-BDNF	SantaCruz, sc-65513	1:500
anti-GAPDH	Abcam, ab8245	1:3000
anti-Actin	Sigma Aldrich, A3853	1:2000
anti-Actin	Cell signaling, #4970	1:3000

Figure 5E. **Gel 1**

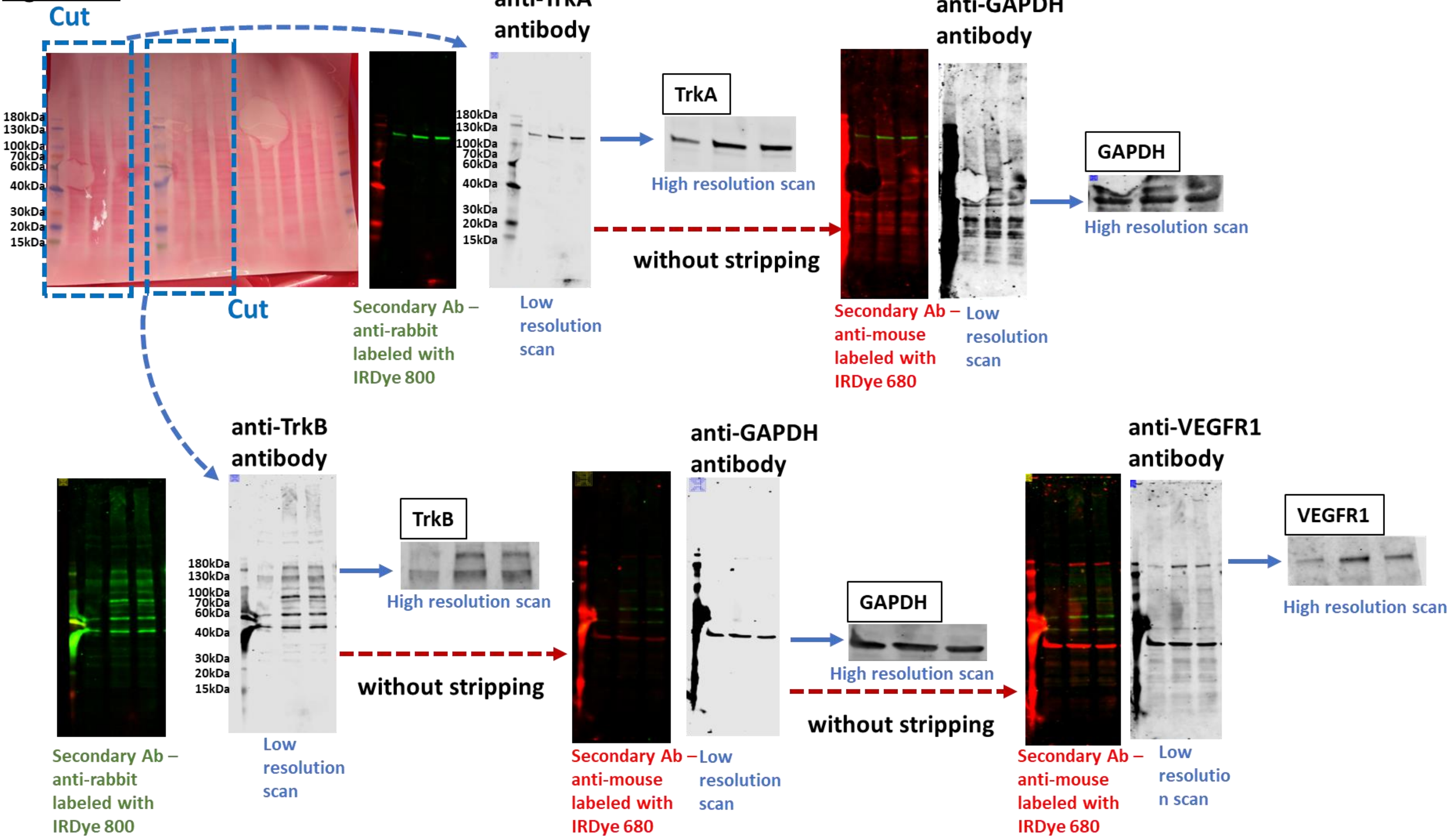

Figure 5E.

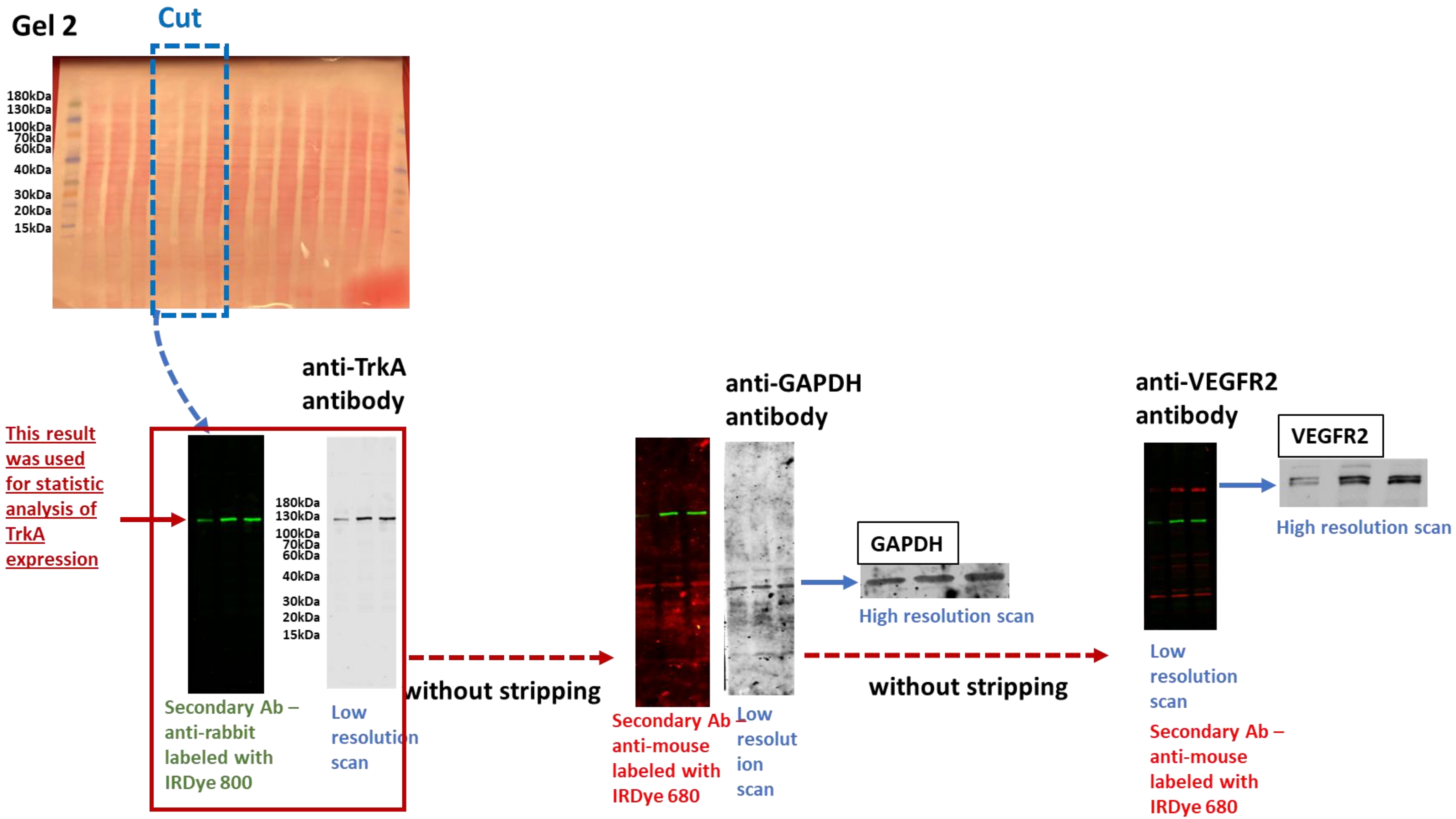

Gel 2
Cut
180kDa
130kDa
100kDa
70kDa
60kDa
40kDa
30kDa
20kDa
15kDa
anti-TrkA antibody
This result was used for statistic analysis of TrkA expression
180kDa
130kDa
100kDa
70kDa
60kDa
40kDa
30kDa
20kDa
15kDa
Secondary Ab – anti-rabbit labeled with IRDye 800
Low resolution scan
without stripping
anti-GAPDH antibody
GAPDH
High resolution scan
Secondary Ab – anti-mouse labeled with IRDye 680
Low resolution scan
without stripping
anti-VEGFR2 antibody
VEGFR2
High resolution scan
Low resolution scan
Secondary Ab – anti-mouse labeled with IRDye 680

Figure 6C.

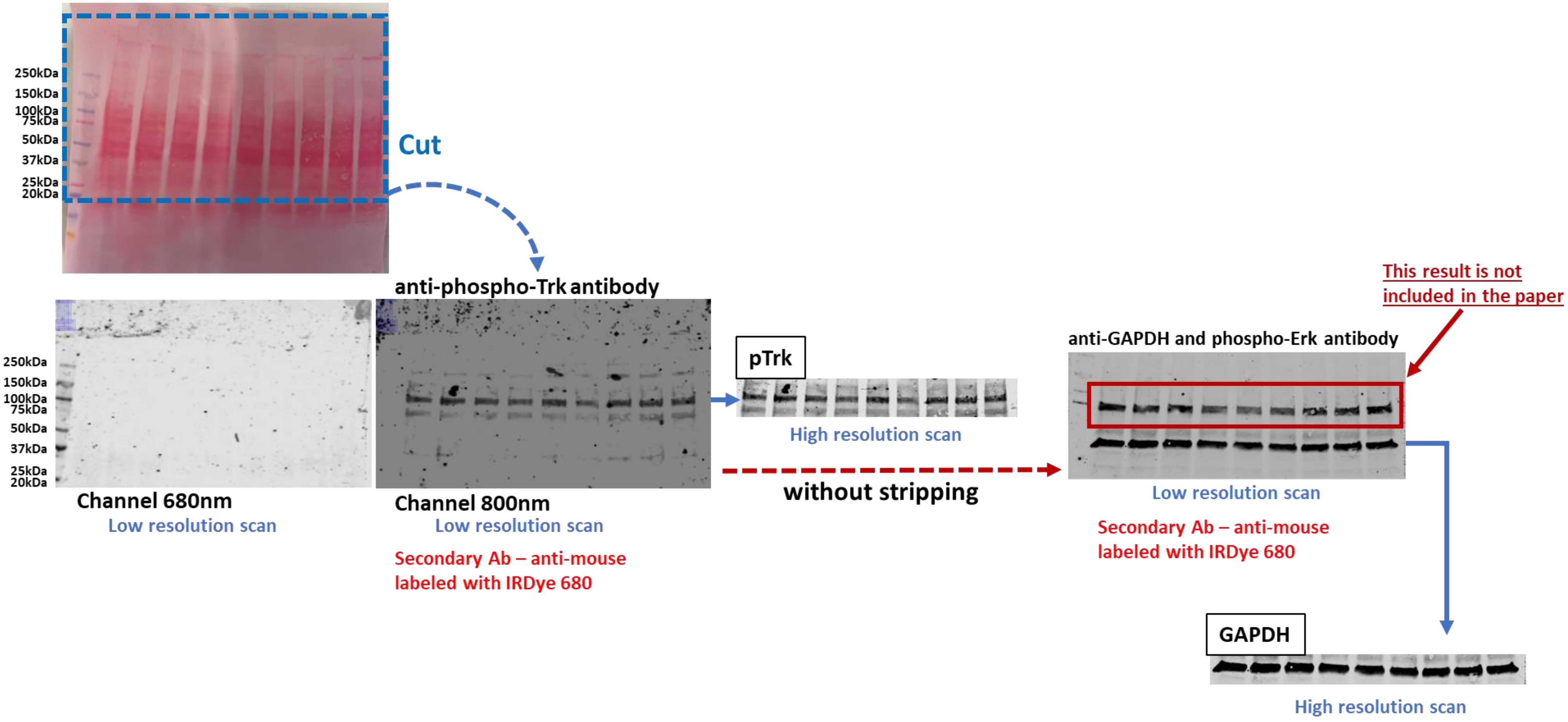
250kDa
150kDa
100kDa
75kDa
50kDa
37kDa
25kDa
20kDa
Cut
anti-phospho-Trk antibody
Channel 680nm
Low resolution scan
Channel 800nm
Low resolution scan
Secondary Ab – anti-mouse
labeled with IRDye 680
pTrk
High resolution scan
without stripping
This result is not
included in the paper
anti-GAPDH and phospho-Erk antibody
Low resolution scan
Secondary Ab – anti-mouse
labeled with IRDye 680
GAPDH
High resolution scan

Figure 6C.

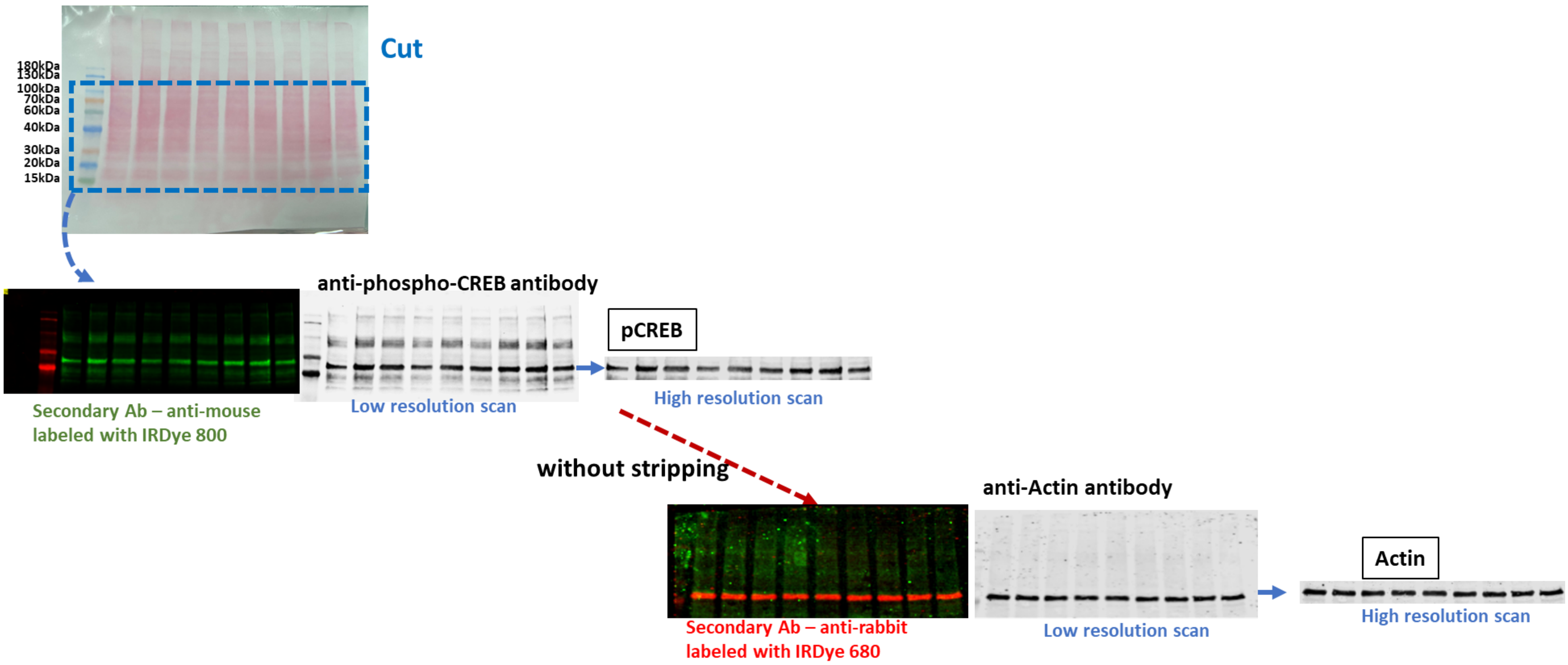
Cut
180kDa
130kDa
100kDa
70kDa
60kDa
40kDa
30kDa
20kDa
15kDa
anti-phospho-CREB antibody
pCREB
Secondary Ab – anti-mouse labeled with IRDye 800
Low resolution scan
High resolution scan
without stripping
anti-Actin antibody
Actin
Secondary Ab – anti-rabbit labeled with IRDye 680
Low resolution scan
High resolution scan

Figure 7C.

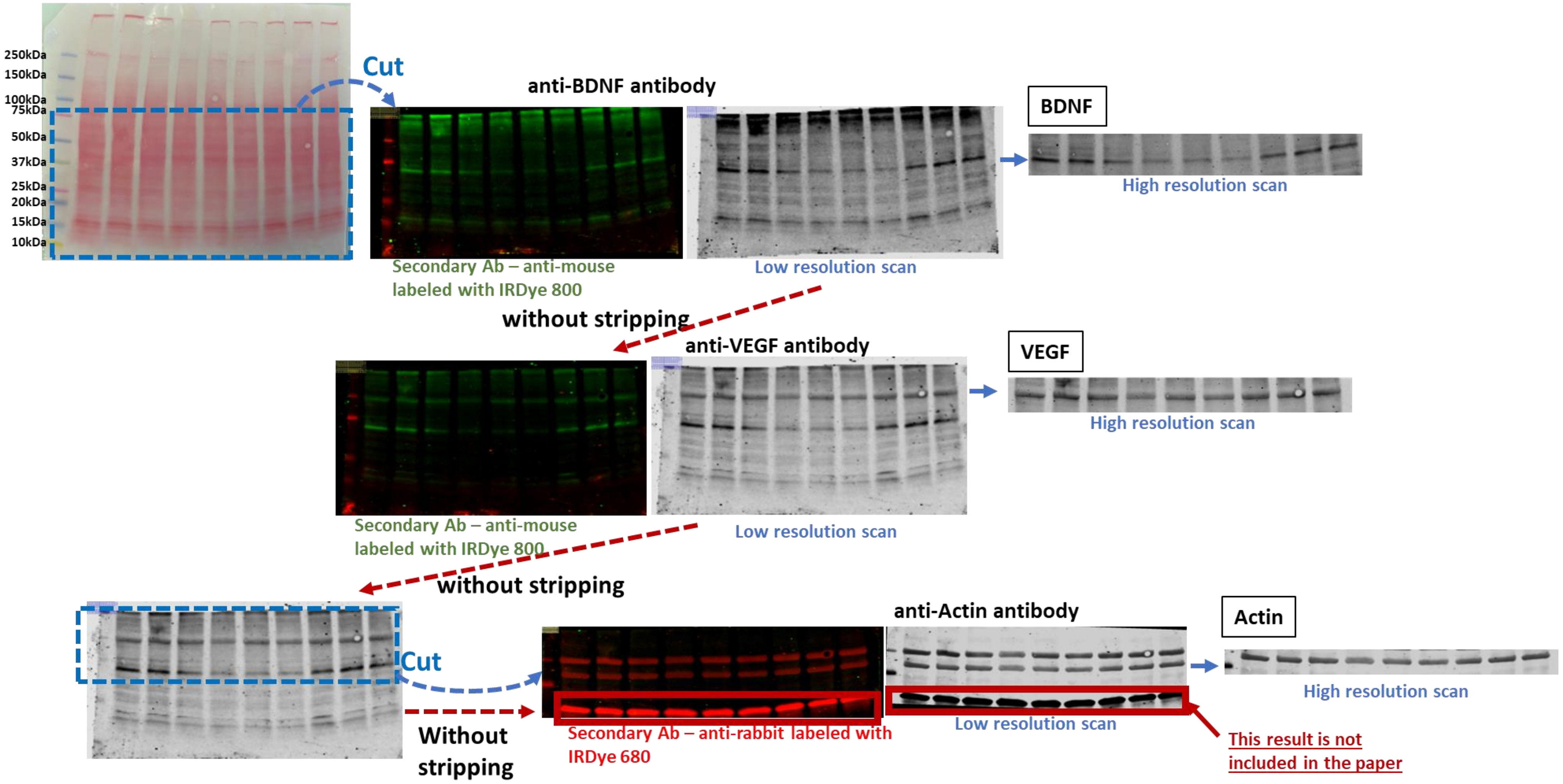

250kDa
150kDa
100kDa
75kDa
50kDa
37kDa
25kDa
20kDa
15kDa
10kDa
Cut
anti-BDNF antibody
BDNF
High resolution scan
Secondary Ab – anti-mouse labeled with IRDye 800
Low resolution scan
without stripping
anti-VEGF antibody
VEGF
High resolution scan
Secondary Ab – anti-mouse labeled with IRDye 800
Low resolution scan
without stripping
anti-Actin antibody
Actin
Cut
High resolution scan
Without stripping
Secondary Ab – anti-rabbit labeled with IRDye 680
Low resolution scan
This result is not included in the paper
